# Targeting hedgehog signaling reduces self-renewal in embryonal rhabdomyosarcoma

**DOI:** 10.1038/onc.2015.267

**Published:** 2015-07-20

**Authors:** S Satheesha, G Manzella, A Bovay, E A Casanova, P K Bode, R Belle, S Feuchtgruber, P Jaaks, N Dogan, E Koscielniak, B W Schäfer

**Affiliations:** 1 Department of Oncology and Children's Research Center, University Children's Hospital, Zurich, Switzerland; 2 Institute of Surgical Pathology, University Hospital Zurich, Zurich, Switzerland; 3 Department of Oncology/Hematology/Immunology, Olgahospital, Klinikum Stuttgart, Stuttgart, Germany

## Abstract

Current treatment regimens for rhabdomyosarcoma (RMS), the most common pediatric soft tissue cancer, rely on conventional chemotherapy, and although they show clinical benefit, there is a significant risk of adverse side effects and secondary tumors later in life. Therefore, identifying and targeting sub-populations with higher tumorigenic potential and self-renewing capacity would offer improved patient management strategies. Hedgehog signaling has been linked to the development of embryonal RMS (ERMS) through mouse genetics and rare human syndromes. However, activating mutations in this pathway in sporadic RMS are rare and therefore the contribution of hedgehog signaling to oncogenesis remains unclear. Here, we show by genetic loss- and gain-of-function experiments and the use of clinically relevant small molecule modulators that hedgehog signaling is important for controlling self-renewal of a subpopulation of RMS cells *in vitro* and tumor initiation *in vivo*. In addition, hedgehog activity altered chemoresistance, motility and differentiation status. The core stem cell gene NANOG was determined to be important for ERMS self-renewal, possibly acting downstream of hedgehog signaling. Crucially, evaluating the presence of a subpopulation of tumor-propagating cells in patient biopsies identified by GLI1 and NANOG expression had prognostic significance. Hence, this work identifies novel functional aspects of hedgehog signaling in ERMS, redefines the rationale for its targeting as means to control ERMS self-renewal and underscores the importance of studying functional tumor heterogeneity in pediatric cancers.

## Introduction

Rhabdomyosarcoma (RMS) comprises of a heterogeneous set of neoplasms that possess features of halted skeletal muscle differentiation and is the most common pediatric soft tissue cancer. The two major histological subtypes, embryonal (ERMS) and alveolar, differ in their molecular cytogenetic profiles, clinical presentations and prognosis. ERMS accounts for about 70% of RMS cases and possesses a relatively more complex genomic landscape with frequent alterations within the RAS pathway.^1, 2, 3^ Currently, pre-treatment histology and initial clinical presentation guide risk stratification to determine therapy intensity. Although a majority of ERMS patients have good prognosis, clinical benefit from current treatments has reached a plateau and prognosis is dismal for high-risk metastatic ERMS patients.^2^ Therefore, there is an urgent need to implement rationally selected targeted treatment options to reduce rate of relapse, therapy burden and improve clinical outcome.

Hedgehog pathway, a master developmental signaling system, is commonly activated in sporadic ERMS.^4, 5, 6, 7^ Canonical hedgehog pathway is a ligand-activated signaling system with three ligand variants—Sonic (SHH), Indian (IHH) and Desert hedgehog (DHH). The secreted ligands bind to the extracellular domain of the Patched (PTCH) receptor leading to the release of the receptor Smoothened (SMO). SMO then translocates to the primary cilium to activate the downstream signaling cascade that involves relieving inhibition of Suppressor of Fused (SUFU) on activity of the GLI transcription factors. The ligands available for activation can be titrated by the transmembrane Hedgehog-interacting protein (HHIP). There are three GLI transcription factors, of which GLI1 is the most potent transactivator. Low-level gains in the GLI1 genomic region have been noted in ERMS patients.^5^ The expression of GLI target genes, which include components of the hedgehog pathway such as GLI1, PTCH1 and HHIP, can be used to study pathway activation status.^8, 9^

Recent data suggest that ERMS is a hierarchically organized tumor.^10, 11, 12^ At present, little is known about the pathways used to maintain self-renewal and tumorigenic properties of ERMS tumor-propagating cells (TPCs). In the present study, using small molecules and various genetic approaches, we show that hedgehog signaling modulates ERMS TPC features of self-renewal and tumor initiation. We describe additional novel roles played by this pathway in determining ERMS chemoresistance, invasion and differentiation, and identify NANOG as a functionally important self-renewal gene that could be downstream of the pathway, previously unknown in any soft tissue sarcoma. Importantly, we show that functional intra-tumoral heterogeneity identified by the presence of hedgehog-active TPC markers in ERMS patients is clinically relevant.

## Results

### Hedgehog signaling is necessary for ERMS self-renewal and efficient tumorigenesis

**Figure 1:**
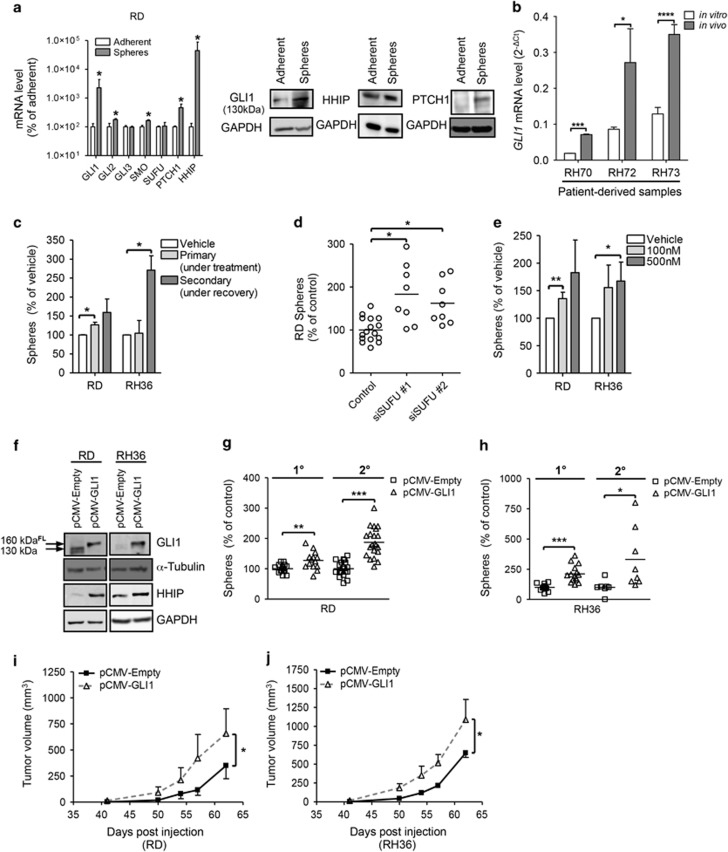
Activation of hedgehog signaling increases self-renewal and tumorigenicity of ERMS cells. (**a**) Left panel: Expression levels of hedgehog signaling components in RD spheres (*n*=6; *N*=2) compared with adherent monolayer cultures (*n*=9; *N*=3) by quantitative PCR (Log_10_ scale). Right panel: Western Blot anaysis showing expression of indicated proteins in RD adherent and sphere cells. (**b**) GLI1 RNA expression levels (relative to HMBS) in patient-derived samples when grown as xenografts (‘*in vivo*' *n*=6, *N*=2) or dissociated and cultured as adherent cells (‘*in vitro*' *n*=6, *N*=2) determined by qPCR. (**c**) Sphere-initiation capacity of ERMS cells treated with hedgehog agonist SAG1.3 (500 nM) every 48 h (three rounds) during primary sphere formation and thereafter plated for secondary sphere formation in normal sphere media (*n*=9; *N*=3). (**d**) Sphere formation after siRNA-mediated knockdown of SUFU (10 nM) in RD adherent cells compared with scrambled control siRNA (*N*=2). (**e**) Sphere formation following 48 h treatment of ERMS adherent cultures with SAG1.3 (*n*=5; *N*=5). (**f**) Western blot analysis of indicated proteins in ERMS stable cell lines. Primary (1°) and secondary (2°) sphere formation measured in ERMS stable lines (**g**: RD; *N*=3 and **h**: RH36; *N*=3). (**i**) Tumor growth rate of RD-based stable lines pCMV-Empty (*n*=6/6) and pCMV-GLI1 (*n*=5/5) injected orthotopically in NOD/SCID mice. (**j**) Tumor growth rate of RH36-based stable lines pCMV-Empty (*n*=2/6) and pCMV-GLI1 (*n*=4/6) injected orthotopically in NOD/SCID mice. Error bars in **i** and **j** represent s.e.m. Each data point in the scatter plots represents a technical replicate with the line drawn at the mean. In bar graphs, data represent mean±s.d. *P<0.05; **P<0.01; ***P<0.001; ****P<0.0001. FL, full-length.

We analyzed the expression of hedgehog pathway components in ERMS sphere cultures that are enriched in de-differentiated, self-renewing and highly tumorigenic cells.^11^ Quantitative PCR (qPCR) analysis revealed that the expression of hedgehog target genes was upregulated in ERMS spheres (Figure 1a and Supplementary Figures 1a and c) and xenografts from ERMS cell lines and patient-derived tumors (Figure 1b and Supplementary Figures 1d and f) compared with adherent cultures. The addition of hedgehog agonist SAG1.3 during primary sphere formation led to enhanced secondary sphere formation (RD: +50% and RH36: +170% Figure 1c) indicating that activating the hedgehog pathway could increase self-renewal in ERMS cells. Similarly, the use of two independent siRNAs against SUFU led to significantly increased sphere-initiating ability (Figure 1d and Supplementary Figures 1g and i). To exclude any extraneous effects of sphere media components on hedgehog pathway activation, ERMS adherent cells were treated with SAG1.3 prior to plating in sphere media. Again, treatment led to a dose-dependent increase in sphere initiation without affecting cell cycle profile or viability (Figure 1e and Supplementary Figures 1j and l).

We performed single cell cloning of RD cell line that showed heterogeneous expression of GLI1 to isolate subpopulations with varying levels of hedgehog pathway activity. All the 29 clones analyzed also showed heterogeneous GLI1 expression (data not shown), indicating that the pathway is dynamically controlled. We performed sphere-initiation studies using two clones (Clones E8 and H3) that had fewer GLI1^high^ cells than bulk RD cells and Clone F2 that was enriched in GLI1^high^ cells (Supplementary Figures 2a and b). Clone E8 and Clone H3 had significantly lower spherogenicity (by 50% and 40%, respectively) and Clone F2 had increased sphere-initiating capacity (+30% Supplementary Figure 2c) highlighting that a population-intrinsic level of hedgehog signaling could determine self-renewal capacity.

To study the role of hedgehog pathway activation in more detail, we generated stable cell lines that overexpressed full-length GLI1 (pCMV-GLI1). At the endogenous level, we could detect the more stable shorter isoform of GLI1 (~130 kDa; Figure 1f) reportedly derived from the 160kDa full-length protein.^13^ pCMV-GLI1 cells showed an increased expression of downstream targets, PTCH1 and HHIP, compared with the control (Figure 1f and Supplementary Figures 2d and e). We also found the expression of PGDFRA, a known hedgehog target gene that was previously implicated in ERMS biology,^14, 15^ to be increased (Supplementary Figures 2d and e). pCMV-GLI1 cells possessed enhanced primary sphere-forming and colony-forming abilities (Figures 1g and h and Supplementary Figure 2f). When plated for secondary sphere formation, the relative increase in sphere-initiation capacity became more apparent (RD: +87% and RH36: +230%) indicating improved self-renewal. Importantly, pCMV-GLI1 cells displayed significantly faster tumor growth rate when injected orthotopically in NOD/SCID mice (Figures 1i and j). The xenografts retained GLI1 overexpression (Supplementary Figure 2g) and were confirmed to be of ERMS histotype (Supplementary Figure 3). Taken together, the active hedgehog pathway in ERMS cells leads to higher self-renewal and increased tumor-initiating capacity.

**Figure 2:**
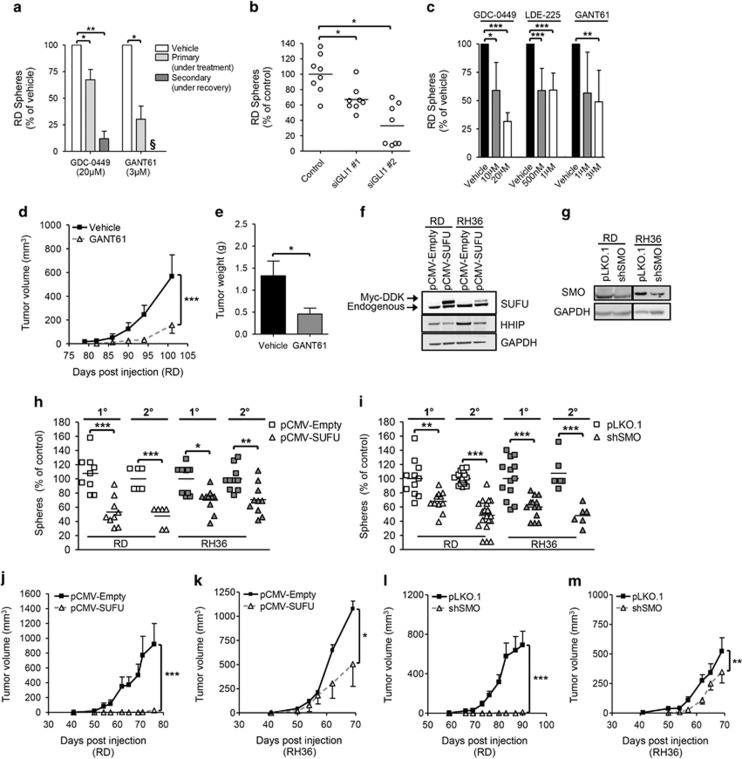
Inhibition of hedgehog signaling decreases self-renewal and tumorigenicity of ERMS cells. (**a**) Sphere-initiation capacity of RD cells treated with small-molecule inhibitors GDC-0449 or GANT61 every 48 h (three rounds) during primary sphere formation and further plated for secondary sphere formation in normal sphere media (*n*=9; *N*=3). **§** No viable cells were recovered for secondary sphere formation. (**b**) Sphere formation measured following siRNA (10 nM) mediated GLI1 knockdown in RD adherent cells (*n*=6; *N*=2). (**c**) Sphere-formation ability of RD adherent cells after 48 h treatment with hedgehog inhibitors (*n*=12, *N*=6 for GDC-0449 and GANT61; *n*=6, *N*=2 for LDE-225). Tumor growth rate (**d**) and tumor weight (**e**) of RD cells pre-treated *in vitro* with GANT61 (3μM) (*n*=5 per condition). Western blot analysis of indicated proteins in stable ERMS lines overexpressing tagged SUFU (**f**; Myc-DDK) and knockdown of SMO (**g**). Primary (1°) and secondary (2°) sphere formation measured in ERMS stable lines (**h**: *N*=2 per cell line; **i**: *N*=2-4 per cell line except 2° sphere formation). Tumor growth kinetics of hedgehog inhibited pCMV-SUFU (**j**: RD; *n*=3/5 and **k**: RH36; *n*=3/6) and control pCMV-Empty (**j**: RD, *n*=5/5 and **k**: RH36, *n*=2/6) cells in NOD/SCID mice. Tumor growth kinetics of hedgehog inhibited shSMO (**l**: RD, *n*=3/7 and **m**: RH36, *n*=6/7) and control pLKO.1 (**l**: RD, *n*=7/7 and **m**: RH36, *n*=7/7) cells in NOD/SCID mice. Error bars in **d**, **e**, **j**–**m** represent s.e.m. In bar graphs, data represent mean±s.d. *P<0.05; **P<0.01; ***P<0.001.

Next, we inhibited the hedgehog pathway both pharmacologically and genetically. ERMS cells treated with SMO inhibitor GDC-0449 or GLI inhibitor GANT61 during primary sphere formation showed decreased sphere numbers (Figure 2a and Supplementary Figure 4a). GDC-0449 treatment led to an 88% decrease in secondary sphere formation for RD cells and RH36 cells showed no spheres formation. GANT61 treatment was more potent because no viable cells were available for secondary sphere formation from either cell line. Using two independent siRNAs against GLI1 in adherent cells significantly decreased sphere initiation (Figure 2b and Supplementary Figures 4b and c). Next, cells were treated with inhibitors (also including the SMO inhibitor LDE-225) under adherent conditions and then plated for sphere formation. Dose-dependent decrease in sphere initiation was observed with all drugs (Figure 2c and Supplementary Figure 4d). Treatments did not alter cell cycle profile or viability status of the cells (Supplementary Figures 4e and h). Pre-treatment of RD cells with GANT61 *in vitro* led to slower tumor initiation *in vivo* owing to reduced hedgehog pathway activity at the time of engraftment (Figures 2d and e and Supplementary Figures 4i–k).

To study the long-term effects of inhibition, we generated stable cell lines that overexpressed SUFU (pCMV-SUFU) to inhibit GLI activity directly or expressed shRNA against SMO (shSMO) to inhibit the canonical ligand-based hedgehog signaling. Both inhibitory systems led to decreased target gene expression (Figures 2f and g and Supplementary Figures 5a and e). Adherent colony-forming ability, sphere initiation and renewal were markedly decreased by either hedgehog-inhibition strategies (Figures 2h and i and Supplementary Figures 5f and g). Although no significant changes occurred in proliferation or cell cycle profiles (Supplementary Figures 5h and n and data not shown), significant decrease in tumor growth kinetics was observed *in vivo* (Figures 2j–m). Impressively, RD cells showed no palpable tumor growth in the majority of hedgehog-inhibited xenografts at the time when the controls reached maximum allowed tumor volumes. While tumor initiation rate was 100% for control cells, only three out of five mice and three out of seven mice injected with pCMV-SUFU and shSMO cells, respectively, eventually developed tumors. Taken together, the inhibition of hedgehog pathway reduces *in vitro* self-renewal and *in vivo* tumor initiation.

Pathway activation seemed to be largely ligand-based because inhibition of either receptor-mediated or GLI-based hedgehog signaling led to similar and comparable effects on self-renewal and tumorigenesis. Accordingly, we found increased expression of hedgehog ligands DHH and IHH in RH36 spheres and xenografts (Supplementary Figures 6a and c), and of DHH in RD cells (Supplementary Figures 6b and d) compared with adherent cultures. Also, patient-derived xenograft (PDX) samples showed higher expression of both (RH70) or IHH ligand (RH73) *in vivo* (Supplementary Figures 6e and f). DHH and IHH were also found to be the most commonly expressed hedgehog ligands in ERMS patient tumors, with on average a higher expression of DHH (Supplementary Figures 6g and h). Surprisingly, SHH was expressed only in a minority of tumor biospies and not at all in adherent cell lines, sphere cultures, xenografts or murine skeletal muscle. Additionally, using species-specific qPCR probes, we could determine that ligand-based signaling was occurring in an autocrine manner with a minor inverse-paracrine contribution from the stroma within xenografts (Supplementary Figures 6k and n). Therefore, ligand-based hedgehog signaling is active in ERMS and seems to increase under conditions of self-renewal and *in vivo* tumorigenesis and, importantly, is necessary for TPC functionality.

### Hedgehog signaling alters chemoresistance, differentiation status and cell motility of ERMS cells

TPCs might also be responsible for tumor recurrence by being more resistant to chemotherapeutic treatments.^16^ To test this notion, we treated our stable cells with serial dilutions of irinotecan or doxorubicin that are currently used in clinical management. We observed on average higher IC_50_ values for pCMV-GLI1 cells compared with a control indicating that cells with increased hedgehog activity are more resistant (Table 1). Conversely, cells with inhibited pathway were more sensitive to conventional drugs. Interestingly, treatment of wild-type RD cells with increasing doses of irinotecan also enhanced sphere-initiating capacity, which could be rescued by combined treatment with hedgehog inhibitor LDE-225 (Supplementary Figures 7a and b). This implies that high-dose chemotherapy treatment currently used in clinical management could enrich for hedgehog-active TPCs.

**Figure 3:**
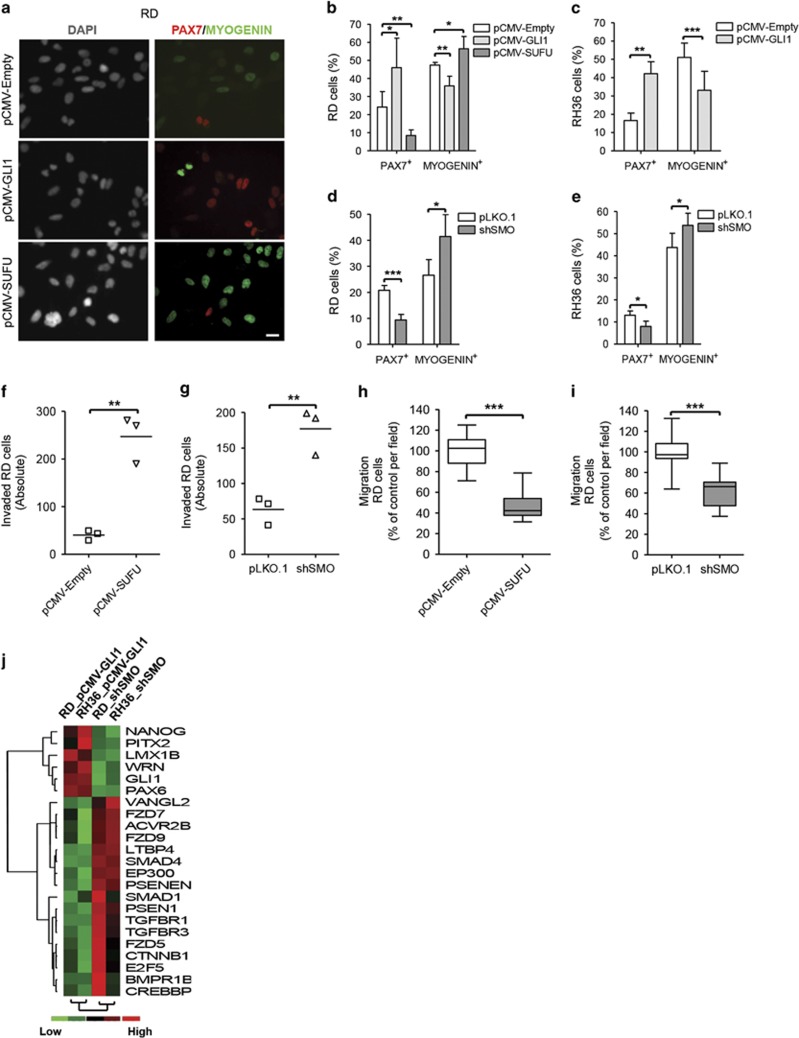
Hedgehog signaling alters the differentiation status and motility of ERMS cells. (**a**) Representative images of RD cells stained for PAX7 and MYOGENIN expression. All images were taken at × 400 magnification. Scale bar represents 20 μm. (**b** and **d**) Quantification of percentage of PAX7- or MYOGENIN-positive RD cells normalized to DAPI-stained nuclei counted per viewing field, using ImageJ (*n*=4). (**c** and **e**) Quantification of PAX7- or MYOGENIN-positive RH36 cells (*n*=5). (**f** and **g**) Total number of RD cells that could invade through matrigel-coated porous membrane filter towards a growth serum gradient over 48 h (*n*=3; *N*=3). (**h** and **i**) Relative migration of RD cells across porous membrane filter towards a growth serum gradient over 48 h (*n*=15; *N*=3). (**j**) Non-supervised hierarchical clustering of genes positively and negatively regulated by the hedgehog pathway common to both RD and RH36 cell lines. Each column represents the average RNA expression fold change for the labelled genes within the hedgehog-modulated stable cell line made relative to its respective empty vector control (*n*=2; *N*=2). *P<0.05, **P<0.01, ***P<0.001. Data represent mean±s.d.

Next, we evaluated the effect of hedgehog signaling on ERMS differentiation. Expression of PAX7 is highest in muscle stem cells, while committed muscle progenitor cells express MYOGENIN. Therefore, expression of these proteins provides a convenient readout to assess the differentiation status. Indeed, the expression of these markers was mutually exclusive also in ERMS cells indicating that the differentiation programs present during normal myogenesis are also active in the pathological state (Figure 3a and Supplementary Figure 7c). pCMV-GLI1 cells possessed increased PAX7^+^ cells and concomitantly fewer MYOGENIN^+^ cells (Figures 3a–c and Supplementary Figure 7c), whereas inhibition of the pathway induced differentiation as evidenced by a reduction in the percentage of PAX7^+^ and gain in MYOGENIN^+^ cells (Figures 3a and Supplementary Figures 7d and e). Treatment with small molecule modulators induced similar alterations in the differentiation status (Supplementary Figures 7f and i). Furthermore, significant increase in the expression of terminal muscle differentiation markers CKM and MYL1 was noted upon long-term treatment with GANT61 (Supplementary Figure 7j). These data suggest that activation of hedgehog signaling confers a more stem-like state, whereas pathway inhibition induces differentiation. We could not find significant co-localization of PAX7 and GLI1 expression (data not shown) indicating that PAX7 may not be a direct target of GLI1. Rather, hedgehog pathway activation might induce de-differentiation by reducing the transcriptional activity of pro-differentiation muscle regulatory factors.^17^

A previous study evaluating *in vivo* tumor heterogeneity in a zebrafish model of ERMS reported that the Myogenin-expressing (differentiated) compartment had higher invasiveness.^12^ Concordantly, we observed that the hedgehog-inhibited cells possessed increased ECM invasion capacity (Figures 3f and g and Supplementary Figure 8a). This effect was cell autonomous because coating the membrane filter with gelatin did not alter the results (Supplementary Figure 8a). Surprisingly, in the absence of a basement membrane matrix, the differentiated cells had much lower migratory ability (Figures 3h and i and Supplementary Figure 8b), indicating that matrix adhesion probably has an important role in determining cell motility in ERMS.

### NANOG is functionally important for ERMS self-renewal

To identify genes that could be regulated by the hedgehog pathway in ERMS cells, we used a stem cell-focused qPCR-based screening approach interrogating 162 genes associated with developmental pathways and the stem cell phenotype. We found 147 genes to be reliably expressed (Ct value<35), of which 142 were common to both cell lines. Non-supervised hierarchical clustering identified genes either positively or negatively modulated by hedgehog signaling (Figure 3j). In general, we found more genes to be negatively regulated by the hedgehog signaling and among them were several components of TGF-β, Wnt and Notch (in RD cells; data not shown) signaling pathways. Interestingly, expression of the stem cell transcription factor NANOG was positively regulated by the hedgehog pathway in both ERMS cell lines studied. NANOG is a homeodomain-containing transcription factor essential for establishing pluripotency^18^ with a known function in TPC maintenance in many adult cancers.^19^ It has already been characterized as a GLI target gene in neural stem cells, medulloblastoma and glioblastoma neurospheres.^20, 21^ Therefore, we chose to further study its role in ERMS.

**Figure 4:**
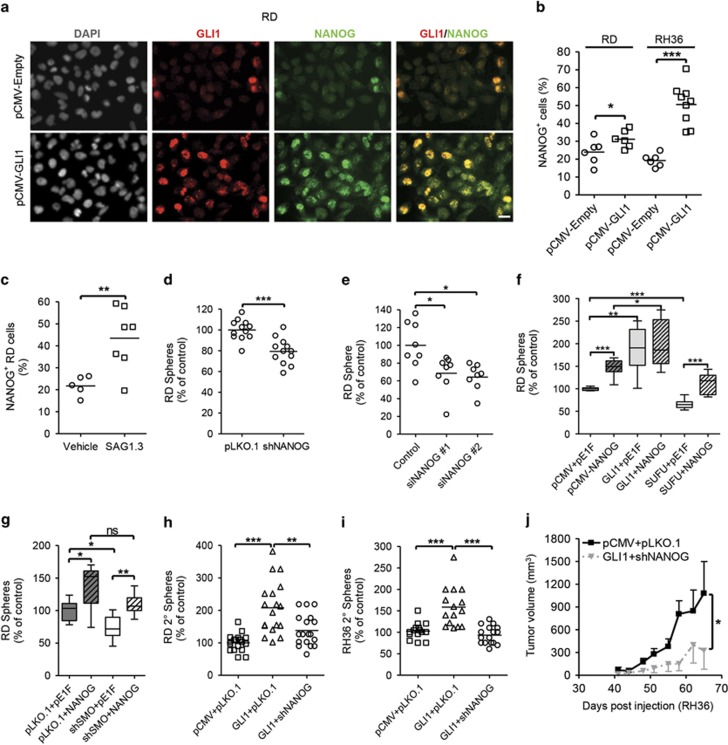
Nanog is a functionally important target gene of hedgehog pathway in ERMS. (**a**) Representative images of RD cells co-stained for GLI1 and NANOG expression. All images were taken at × 400 magnification. Scale bar represents 20 μm. Quantification, using ImageJ, of NANOG-expressing cellular compartments normalized to DAPI-stained nuclei per viewing field in ERMS stable lines (**b**; *N*=2) and RD cells treated with SAG1.3 (500 nM) for 48 h (**c**; *N*=2). (**d**) Sphere formation in RD cells with stable knockdown of NANOG (shNANOG; *N*=3). (**e**) Sphere formation measured following siRNA (10 nM) mediated NANOG knockdown in RD adherent cells (*N*=2). (**f** and **g**) Primary sphere formation upon transient overexpression of NANOG in RD cells (*n*=12; *N*=2). Data represent mean±s.d. Secondary sphere formation in RD (**h**) and RH36 (**i**) rescue systems (*N*=3). (**j**) Tumor growth rate of RH36 cells in NOD/SCID mice (*n*=6/6 per cell line). Error bars represent s.e.m. *P<0.05, **P<0.01, ***P<0.001. ns, not significant.

First, we confirmed alterations in NANOG expression noted in the screen on additional samples (Supplementary Figures 9a and d). Next, ERMS cells were co-immunostained for GLI1 and NANOG. The expression of both proteins was found to be heterogeneous and strictly co-localized in both cell lines and primary cells from three PDX samples (Figure 4a and Supplementary Figures 9e and f). Upon GLI1 overexpression or SAG1.3 treatment, the percentage of NANOG-expressing cells increased significantly (Figures 4b and c and Supplementary Figures 10a and c). NANOG expression was higher in sphere cultures and xenografts than adherent cultures (Supplementary Figures 10d and g). These data indicate that NANOG expression correlates with hedgehog pathway activity, both in ERMS cells and patient samples, implying that it could be a target gene of the pathway in ERMS similar to observations in other cancers.

We also reduced NANOG expression in RD cells using transient and stable genetic means and both approaches led to decreased sphere formation (Figures 4d and e). Transient overexpression of NANOG significantly improved spherogenicity (Figures 4f and g). Importantly, ectopic NANOG expression in hedgehog-inhibited cells rescued the lowered self-renewal ability back to the level of controls, indicating that NANOG could act epistatic to hedgehog pathway (Figures 4f and g). We also noted that alteration in NANOG expression led to a concordant change in the expression of GLI1 (Supplementary Figures 11a and c), which could be either due to the effect on overall proportion of ERMS stem cell-like population marked by GLI1 expression or the direct modulation of GLI1 expression as previously reported in brain cancers.^21^ Interestingly the expression of PDGFRA was also decreased upon NANOG knockdown (Supplementary Figure 11a). To evaluate the role of NANOG on self-renewal and tumor growth independently, we generated stable rescue lines where NANOG expression was decreased in GLI1 overexpressing cells (GLI1+shNANOG) and corresponding empty vector (pCMV+pLKO.1) or GLI1 overexpression only controls (GLI1+pLKO.1) (Supplementary Figures 11d and e). When both RD and RH36 rescue systems were allowed to form spheres, secondary sphere formation was increased significantly in the GLI1+pLKO.1 cells for both cell lines (RD: +108% and RH36: +59%) and impressively, NANOG knockdown rescued it back to almost control levels (Figures 4h and i). Additionally, the *in vivo* tumor growth rate of GLI1+shNANOG cells was significantly lower than control cells (Figure 4j and Supplementary Figures 11f and g). Taken together, NANOG emerges as a functionally important gene for TPC properties in ERMS that could act downstream of the hedgehog signaling.

### GLI1 and NANOG expression has prognostic value for ERMS patients

**Figure 5:**
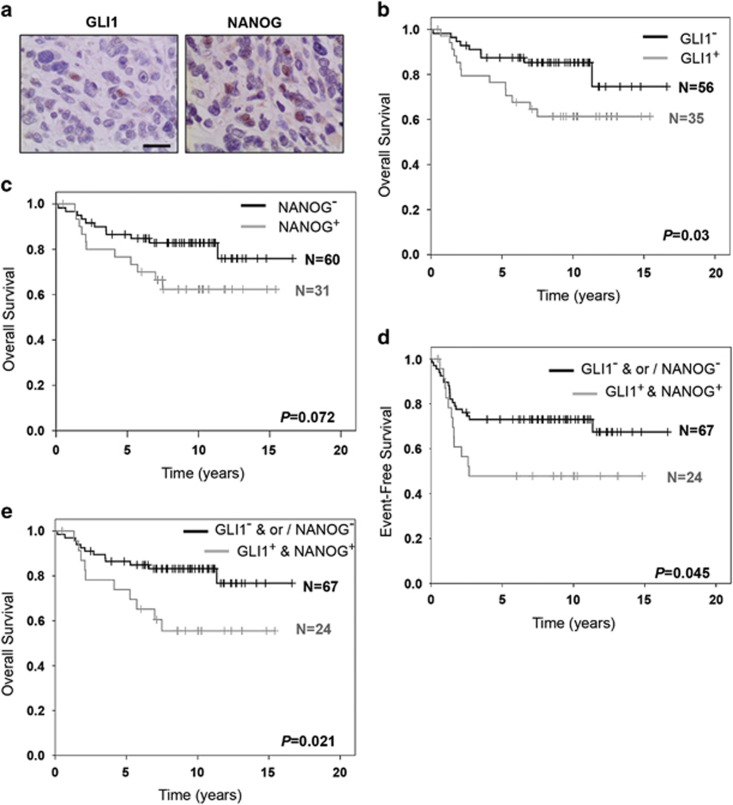
Presence of GLI1+ and NANOG+ compartment predicts adverse patient survival. (**a**) Representative images of immunohistochemical staining for GLI1 and NANOG within an ERMS patient tumor core. All images were taken at × 400 magnification. Scale bar represents 20 μm. Kaplan–Meier curve representing overall survival of 91 ERMS patients either negative (black line) or positive (grey line) for GLI1 (**b**) or NANOG (**c**) alone. Kaplan–Meier curve representing event-free survival (**d**) and overall survival (**e**) of 91 ERMS patients determined to be negative (black line) for GLI1 and/or NANOG (‘GLI1^-^ & or / NANOG^-^') or positive (grey line) for both GLI1 and NANOG expression (‘GLI1^+^ & NANOG^+^'). The *P*-values were generated using log-rank test.

Finally, we evaluated whether the expression of GLI1 and NANOG is clinically relevant. To this end, we performed a double-blind analysis of GLI1 and NANOG expression in a previously described set of tissue microarrays (TMA) with multiple tumor cores from 116 ERMS patient samples using immunohistochemistry.^22^ Reliable protein expression status was obtained for 91 patients, most of whom were negative for both proteins. However, patients positive for one were in 80% of the cases also positive for the other. We observed that only tumor cells expressed GLI1 and NANOG and, importantly, the expression was heterogeneous as seen in cell cultures (Figure 5a and Supplementary Figure 11h). Correlation with clinical data revealed that the expression of GLI1 alone could predict significantly worse overall survival and a similar trend was observed for NANOG status (Figures 5b and c). These patients also tended to have worse event-free survival although the data did not reach statistical significance (Supplementary Figures 11i and j). Importantly, it was only when the patients were distinguished based on the presence of GLI1^+^ and NANOG^+^ cellular sub-populations within their tumors that we observed statistically significant worse event-free and overall survival (Figures 5d and e). The distribution of patient- and tumor-related parameters was similar among patient subgroups. Chi-square tests revealed no significant differences between the groups (data not shown). Owing to low patient numbers, it was not possible to assess whether GLI1 and/or NANOG expression could be used as independent prognostic markers. Nevertheless, our analysis reveals that intra-tumoral heterogeneity represented by the expression of both GLI1 and NANOG can help identify a subset of ERMS patients with worse outcome and therefore is clinically relevant.

## Discussion

Although previous studies have identified hedgehog signaling as a clinically relevant pathway in ERMS,^4, 5^ its functional role in ERMS pathology and particularly its contribution to the hierarchical organization seen in ERMS has not been investigated. Here, we show that hedgehog pathway activity is an important determinant of ERMS ‘stemness' features such as self-renewal and tumor initiation, as previously shown for other malignancies.^23^ Therefore, clinical strategies for using hedgehog inhibitors in ERMS would need to accommodate the conceptual implications of the cancer stem cell model.^24, 25^ For instance, tumor regression may not be an appropriate endpoint to estimate treatment efficacy, because the effect of hedgehog pathway modulation on ERMS pathology was not due to changes in cell cycle, cell viability or proliferation.

Pathway activation seems to be occurring primarily by autocrine secretion of IHH and/or DHH. This is in line with an earlier study ruling out SHH autocrine signaling in ERMS patients.^6^ Pathway inhibition was effective at the level of SMO as well as of GLI, which could avoid emergence of cross talks converging on the GLI-code and resistance mechanisms in the clinics.^9^ Furthermore, we found that modulating the pathway can alter sensitivity to generic drugs. Therefore, combination therapy with hedgehog inhibitors might allow the usage of lowered drug doses to reduce treatment-related morbidity.

Interestingly, our work suggests novel negative feedback mechanisms between hedgehog signaling and key muscle differentiation pathways, Wnt, Notch and TGFβ.^26, 27, 28^ Previous reports show that inhibition of Notch and TGFβ and activation of Wnt could lead to ERMS differentiation.^7, 29, 30^ Interestingly, activation of the Wnt pathway induced differentiation and reduced tumor initiation in a RAS-driven zebrafish ERMS model. The authors also identified the hedgehog inhibitor cyclopamine as one of the top drug candidates from a large-scale small molecular screen that could differentiate ERMS cells and reduce tumor growth *in vivo*.^31^ This study supports our findings and further highlights crosstalk between ‘stemness' pathways that could define ERMS TPC behavior. However, the more differentiated compartments are also likely to have important roles in tumor progression (this study and Ignatius *et al.*^12^).

Previously, PTCH1 mRNA expression in ERMS patients was shown to predict poor outcome.^4^ Although PTCH1 mRNA expression correlates significantly with that of GLI1 mRNA (data not shown and Zibat *et al.*^4^), PTCH1 provides negative feedback cues into the pathway that could obscure the final outcome on cellular self-renewal. Hence, PTCH1 protein expression status may not be a reliable predictor of prognosis. We reported that ERMS patients with CD133^high^ expression have poor overall survival,^11^ but the functional role of CD133 protein is currently unclear. Also, the impact of other potential ERMS TPCs markers (FGFR3 (ref. 32) and MYF5 (ref. 12) on prognosis is unknown. We identify NANOG as a functionally important gene whose expression along with GLI1 could serve as novel prognostic indicators. Importantly, we detected clear GLI1-NANOG co-localization in ERMS PDX cells. However, we were unable to do so on the TMA tumor cores owing to the lack of serial sections. Interestingly, we observed heterogeneous expression of GLI1 and NANOG also within alveolar RMS patient tumor cores but without prognostic significance (data not shown). Therefore, the GLI1-NANOG TPC marking could be important specifically for ERMS patient stratification and further highlights the biological disparity between the two RMS variants.

Expression of transcription factors important for development of neural crest-derived mesenchymal and neural tissues, namely PAX6, PITX2 and LMX1B,^33, 34, 35^ were positively regulated by the hedgehog pathway, whereas myogenic differentiation factors were downregulated. Therefore, GLI1-NANOG^high^ ERMS cells could possess a pre-myogenic multipotent phenotype reminiscent of neural crest or non-myogenic origin for ERMS. This is concordant with recent observations in hedgehog-activated mouse models, wherein ERMS ‘cell of origin' was determined to be from either pre-somitic or non-muscle mesenchyme.^36, 37^ ERMS cells expressing other potential TPC markers have been described to be restricted to the skeletal muscle lineage.^10, 32, 38^ It is therefore possible that different ERMS TPC subpopulations with varying differentiation potentials are concomitantly present within tumors. The hierarchical relationship between these potential compartments and their relative importance for ERMS tumorigenicity is yet to be determined and warrants further research.

It is likely that the hedgehog-active TPC phenotype is a widespread feature because activation of RAS signaling is common in ERMS^1, 3^ and is known to have a positive influence on hedgehog signaling.^39^ Interestingly, also, the loss of p53 can increase GLI-NANOG signaling in stem cells and TPCs of neural origin.^20, 21^ The cell lines used in this study represent these genetic backgrounds (RD: NRAS^*Q61H*^; p53^*R248V*^ and RH36: HRAS^*Q61K*^). Hence, hedgehog-driven targeting could be of broad interest in sporadic ERMS. Our study highlights that phenotypic and functional tumor heterogeneity could have significance for clinical management of ERMS patients and suggests hedgehog inhibition as a treatment strategy aimed at reducing the rate of relapse for a long-term cure.

## Materials and methods

### Patient-derived samples

Early passages of ERMS PDX samples RH70 (SJRHB011_Y), RH72 (SJRHB013_X) and RH73 (SJRHB011_X) were obtained from St. Jude Children's Research Hospital (USA) and previously described in detail in.^1^ All patient tissue specimens used only for RNA extraction were obtained from the Swiss Pediatric Oncology Group (SPOG) Tumor Bank except ZH_ERMS, which was obtained from the Department of Pathology, University Hospital Zurich. The use of SPOG Tumor Bank tissue samples was approved by the Ethical Review Board of Zurich (Ref. No. StV-18/02). Written informed consent was obtained from each patient by the hospital that provided the tissue samples. The TMA used in this study included multiple tumor cores from 149 RMS patients (116 ERMS and 33 alveolar RMS) enrolled in the German soft-tissue sarcoma group (CWS) studies -81, -86, -91 and -96 as previously described.^22^

### Orthotopic xenograft generation

RD (3 × 10^5^cells/mouse) or RH36 (2.5 × 10^5^cells/mouse) were injected into the femoral muscles of one leg of 4–6-week-old NOD.CB17-*Prkdc*^*scid*^ mice (NOD/SCID; Jackson Laboratory, Bar Harbor, ME, USA). Animals were chosen from either sex and were assigned randomly to different groups. Once tumor was palpable, size was determined every 4 days by measuring two diameters (d_1_ and d_2_) in right angles of both legs with a Vernier caliper until tumors reached the maximum allowed volume of 1000 cm^3^ or followed for 120 days. Tumor volumes were calculated using the following formula: V=[4/3 x π x 1/2(d_1_+d_2_)]_injected leg_−[4/3 x π x 1/2(d_1_+d_2_)]_control leg_.

For PDX generation, dissociated cells were resuspended in matrigel (Corning, Amsterdam, Netherlands) at 1 × 10^4^ cells/μl and 100 μl was injected as described above.

Freshly isolated xenografts were stored in RNAlater (Ambion, Huntingdon, Cambridgeshire, UK) for RNA extraction, snap-frozen in liquid N_2_ for protein extraction or fixed in 4% paraformaldehyde for imunohistochemistry. The experiments were conducted in a non-blind manner and approved by the veterinary office of Canton Zurich.

### Tumor dissociation

PDX samples were minced with scalpels and digested using Liberase DH (0.62 WU/ml; Roche, Rotkreuz, Switzerland) in buffer containing 1 × HBSS, 10 mM HEPES, 200 U/ml DNase (Roche) and 1 mM MgCl_2_, at 37 °C for 70 min. The cells were pelleted, resuspended in Dulbecco's modified Eagle's media +10% fetal bovine serum and passed through a cell strainer to remove debris. Primary cells were plated and maintained in standard culture media for experiments.

### Cell culture and treatments

Human ERMS cell lines RD (ATCC, Manassas, VA, USA), RH36, RH18 (both kindly provided by Peter Houghton, St. Jude's Children's Hospital, USA) and TTC442 (kindly provided by Timothy J. Triche, Children's Hospital Los Angeles, USA) were cultured in Dulbecco's modified Eagle's media supplemented with 10% fetal bovine serum, 2 mM L-glutamine and 100 U/ml penicillin-streptomycin. Sphere cultures were maintained as previously described.^11^ The cell lines were authenticated by short tandem repeat analysis and regularly checked for mycoplasma contamination. For sphere formation, equal numbers of cells were plated at clonal density in Ultra-Low attachment plates (Corning). Primary spheres were dissociated using Accutase (Sigma-Aldrich, Buchs, Switzerland) and stained with Trypan Blue solution (Sigma-Aldrich) for counting. Equal numbers of viable sphere cells were plated for secondary sphere formation. For single cell cloning, RD cells were plated in 96-well plates in stringent single cell dilution (0.5 cell/well) in normal adherent culture media. After 16 h, the wells with single cells were marked and followed for viable colony formation. Upon reaching confluency, the cultures were propagated in larger plate formats. Drugs used included SMO inhibitors GDC-0449 and LDE-225 (Selleck, Munich, Germany), GLI inhibitor GANT61 (Tocris, Bristol, UK) and GLI activator SAG1.3 (Calbiochem, San Diego, CA, USA). For IC_50_ measurements of irinotecan (SN-38; Sigma-Aldrich) and doxorubicin (Sandoz, Rotkreuz, Switzerland), cells were plated in quadruplicate in 96-well plates and treated with five-step serial dilutions for 72 h in 10% fetal bovine serum media. Dimethyl sulfoxide (Sigma-Aldrich) was used as vehicle control except for SAG1.3 (diluted in water).

### siRNA transfection

Adherent cells were transfected with Silencer select siRNAs (Ambion, Life technologies) against GLI1 (#1: s5814; #2: s5816), SUFU (#1: s28520; #2: s28521), NANOG (#1: s36649; #2: s36650) or scrambled control (Silencer Negative Control# 2) using Lipofectamine RNAiMAX (Invitrogen, Zug, Switzerland) at a final concentration of 10 nM. Sphere growth was initiated 24 h post transfection.

### Cell viability, proliferation and clonogenic assays

To assess cell viability and proliferation, cells were plated in quadruplicate per condition in 96-well plates. After treatment, viability was measured using WST-1 (Roche). Cell proliferation was measured 24 h post plating using Cell Proliferation ELISA, BrdU (chemiluminescent) assay (Roche). Clonogenic assay was performed as described by Franken *et al.*^40^ In brief, cells were seeded in dilution of 1 cell/μl in six-well plates in normal culture media. Media was changed every 3 days until colonies (>50 cells) were visible. Cells were fixed and stained using with Crystal Violet staining solution (0.5% Crystal Violet and 6% gluteraldehye in water). Colonies were quantified using ImageJ software (version 1.47).

### Quantitative PCR

Normal human skeletal muscle pooled RNA lysate, referred to as AdSkM_P, from five adults (R1234171_P) and individual RNA lysates from three fetal donors (R1244171; Lot # A503105, B505186, A508111) were purchased from (Amsbio, Lugano, Switzerland). Total RNA was extracted using RNeasy Mini Kit (Qiagen, Basel, Switzerland) with RNase-free DNase. Normal murine muscle RNA was extracted from femoral muscle of NOD/SCID mice. Complementary DNA synthesis was carried out using High-Capacity cDNA Reverse Transcription kit (Life Technologies, Zug, Switzerland). QPCR was performed using Taqman mastermix and Gene Expression Assays (Life Technologies; assay IDs are listed in Supplementary Table 1). Absolute and relative expression levels were calculated using the ΔΔCt method and normalized to HMBS (unless otherwise specified) or GAPDH. For screening, RT^2^ Profiler PCR Arrays (Stem Cell Signaling (PAHS-047ZE) and Stem Cell Transcription Factors (PAHS-501ZE)) were purchased from Qiagen. Data analysis was performed using the RT^2^ Profiler PCR Array Analysis web-based software (version 3.5). Non-supervised hierarchical clustering was performed using by dChip.

### Western blotting and immunofluorescence

Total protein was extracted using RIPA buffer (50 mM Tris-Cl, pH 6.8, 100 mM NaCl, 1% Triton X-100, 0.1% SDS) supplemented with Complete Mini Protease Inhibitor Cocktail (Roche). Proteins were separated using NuPAGE gradient SDS–PAGE pre-cast gels (Life Technologies) and detected by chemiluminescence using Amersham ECL Detection reagent (GE Healthcare, Glattbrugg, Switzerland) or SuperSignal West Femto Maximum Sensitivity Substrate (Thermo Scientific, St Leon-Rot, Germany). For immunofluorescence, cells were fixed with 4% paraformaldehyde and incubated over night at 4 °C with primary antibodies. All the antibodies and further details are listed in Supplementary Table 2.

### TMA scoring and data analysis

TMA was evaluated by a senior pathologist (PB). A minimum of two desmin-positive intact cores was required for the patient to be included in the analysis. At least three stained cells were required to label a patient as positive. Tumors from 91 ERMS and 23 alveolar RMS patients provided reliable GLI1 and NANOG expression status. Clinical data were analyzed independently by CWS study member (SF) who was blind to the hypothesis. Statistical analysis was performed using SPSS software (version 21, (IBM, Armonk, NY, USA)) based on all data available up to the cutoff date, 05.04.2013. Differences in survival rates were analyzed using the log-rank test.

### Statistical analysis

Data were analyzed using GraphPad Prism (version 4.03). Significance was calculated using Student's *t*-test (unpaired, two-tailed), and if the variance was found to be significantly different by F-test, then Welch's correction was used. Normal distribution of data was assessed using D'Agostino and Pearson normality test. Mann–Whitney test was used to assess significance when data were non-parametric. Tumor growth curves were compared using two-way analysis of variance with Bonferroni *post hoc* tests. Only animals that showed tumor growth were included in the final analysis. *P*<0.05 was considered significant. The total sample size (‘*n*') and biological replicates (‘*N*') per condition per experiment are indicated in the figure legends. Each experiment was replicated at least twice. Data are represented as mean with s.d. as error bars unless otherwise mentioned. No power analysis was used to pre-determine sample size.

## Figures and Tables

**Table 1:** Hedgehog pathway modulation alters chemoresistance of ERMS cells

*Cell lines*		*Mean absolute IC*_*50*_ *at 72* *h (nM*±*s.d.)*				*Phenotype*	
*Parental*	*Transgenic*	*Irinotecan (SN-38)*^a^	*Relative change in IC*_*50*_^c^^,^^d^	*Doxorubicin*^b^	*Relative change in IC*_*50*_^c^^,^^d^	*Hedgehog pathway activity*	*Relative resistance*
RH36	pCMV-Empty	11±6		157±84		Control	
	pCMV-GLI1	19±15	**↑** 73% (ns)	284±205	**↑** 81% (ns)	Increased	Increased
RD	pCMV-Empty	54±30		186±124		Control	
	pCMV-GLI1	94±55	**↑** 74% (*)	286±249	**↑** 54% (ns)	Increased	Increased
	pCMV-SUFU	37±15	**↓** 31% (ns)	139±135	**↓** 25% (ns)	Decreased	Decreased
RH36	pLKO.1	23±15		221±130		Control	
	shSMO	10±5	**↓** 56% (**)	105±28	**↓** 52% (*)	Decreased	Decreased
RD	pLKO.1	78±36		198±135		Control	
	shSMO	39±16	**↓** 50% (***)	177±121	**↓** 10% (ns)	Decreased	Decreased

Abbreviations: ERMS, embryonal rhabdomyosarcoma.

a *N*=5 for RD-based cell lines; *N*=4 for RH36-based cell lines.

b *N*=4 for all cell lines

.

c ‘**↑**' – IC_50_ increased by; ‘**↓**' – IC_50_ decreased by.

d In parenthesis are presented the statistical significance of the relative difference in the IC_50_ values: ns-not significant; *P<0.05; **P<0.01.
