# Supplementary material for: Targeting hedgehog signaling reduces self-renewal in embryonal rhabdomyosarcoma
Source: Oncogene. 2015 Jul 20;35(16):2020–30. doi: 10.1038/onc.2015.267 (PMC5399168; doi:10.1038/onc.2015.267)
Supplement: Supplementary Materials [file onc2015267x1.doc]

**Supplementary materials**

**Transfection, transduction and generation of stable cell lines**

The plasmids pCMV6-Entry (C-terminal Myc and DDK Tagged) referred to as pCMV-Empty, GLI1-transcript variant 1- Myc-DDK (referred to as pCMV-GLI1), SUFU-Myc-DDK tagged (referred to as pCMV-SUFU) were purchased from Origene. The plasmids pEGIP (26777; referred to as pE1F), and pSin-EF-Nanog-Pur (16578; over-expressing NANOG) were purchased from Addgene. All plasmid transfections were carried out using Lipofectamine® 2000 (Invitrogen) following manufacturer’s instructions. Transduction of MISSION® TRC1.5 shRNA lentiviral particles (Sigma-Aldrich) - control (pLKO.1; SHC001V), TRCN0000014364 (shSMO) and TRCN0000004885 (shNANOG) - was carried out at MOI 1.5 in 1µg/ml Polybrene (Sigma-Aldrich) supplemented media. For stable cell line generation, plasmid transfected cells were continuously cultured in media supplemented 500 µg/ml G-418 antibiotic for 3-4 weeks and thereafter maintained in 100 µg/ml antibiotic media; viral transduced cells were continuously cultured in media supplemented with 1µg/ml Puromycin (Invivogen) for 10 days and thereafter maintained in 0.3 µg/ml antibiotic media. Stable pCMV-Empty and pCMV-GLI1 cells were transduced with pLKO.1 and shNANOG viral particles and selected with Puromycin as described above to generate stable phenotype rescue system. The effect of the expression of the transgene was regularly checked using qPCR and western blotting through the course of the experiments. Occasionally the stable lines were challenged with higher doses of antibiotics to ensure that the cell lines being propagated carried the transgene constructs.

**Trans-well migration and invasion assays**

Migration assays were carried out using BD Falcon™ cell culture inserts (BD Biosciences; 8µm pore size) in 24-well format. For invasion assays, BioCoat™ matrigel-coated inserts (BD Biosciences) or inserts coated manually with gelatin were used. Cells were maintained in 1% FBS media for 4hrs prior to plating and allowed to migrate over 24hrs or invade over 48hrs towards 10% FBS medium. The membrane was fixed with 4% paraformaldehyde (PFA; Carl Roth), cells stained with 0.05% Crystal Violet and visualized using Olympus CX41 microscope. Images were captured from 5 viewing fields across the membrane using INFINITY software (version 6, Lumenera). Cells were counted manually using ImageJ software (version 1.47).

**Cell cycle analysis**

Cells were fixed with ice-cold 70% ethanol and re-suspended in propidium iodide solution (1xPBS, 1% Triton X-100, 100mg/ml RNaseA, 1mg/ml Propidium Iodide solution) just before analysis. For intra-cellular staining, cells were fixed with cold 4% PFA and permeabilized with 0.2% Triton X-100. Antibodies used are indicated in Supplementary Table 2. Flow cytometry was performed on a BD FACS Canto II instrument (BD Biosciences) using BD FACSDiva software. Data was analyzed with FlowJo software (version 7.6.1, TreeStar Inc.).

**Immunohistochemistry**

Cells were visualized using Leica 6000 DM epifluorescence microscope and images were captured using OpenLab software (version 3, Improvision). Cells stained only with conjugated-secondary antibodies were used as controls. Cell count estimation and image analysis was performed using ImageJ. Immunohistochemistry was performed on 3 µm thick sections from blocks of formalin-fixed, paraffin-embedded tissue at the Institute of Surgical Pathology (University Hospital Zurich). The procedure for detecting GLI1 and NANOG expression was optimized by Sophistolab AG (Eglisau, Switzerland) using an array containing a series of normal tissue and cancer tissues as controls. In brief, immunohistochemistry was performed according to manufacturer’s guidelines on Leica BondMax instruments using Refine HRP-Kits (Leica DS9800) and buffer-solutions from Leica Microsystems Newcastle, Ltd. Slides were visualized using Zeiss Axioskop microscope. Images were captured and analyzed using Cell^B^ software (version 3.4; Olympus Soft Imaging Solutions GmbH).

**Supplementary Figure legends**

**Figure 1.** Analysis of ERMS spheres, xenografts and effect of transient activation of hedgehog pathway. mRNA expression of multiple hedgehog signaling components in RH36 (**a**; n=9; N=3 per cell type), TTC442 (**b**; n=6; N=2 per cell type) and fusion-negative RH18 (**c**; n=6; N=2 per cell type) spheres compared to adherent monolayer cultures by qPCR. (**d**) GLI1 mRNA expression levels (relative to HMBS) in indicated cell lines when grown as xenografts (“*in vivo*”; n=6, N=2) or cultured as adherent cells (“*in vitro*”; n=9, N=3) determined by qPCR. PTCH1 (**e**) and HHIP (**f**) mRNA expression levels relative to HMBS in indicated ERMS sample determined by qPCR (for PDX: n=6, N=2 per condition; for cell lines: n=6, N=2 for “*in vivo*” and n=9; N=3 for “*in vitro*”). (**g**) Western blotting for indicated proteins upon siRNA mediated knockdown of SUFU in RD adherent cells. (**h**) Sphere formation measured following siRNA (10nM) mediated SUFU knockdown in RH36 adherent cells (N=2). (**i**) SUFU protein expression in RH36 adherent cells after siRNA-mediated knockdown. Flow cytometry-based cell cycle profile estimation from Propidium Iodide staining of RD (**j**) and RH36 (**k**) cells treated with SAG1.3 for 48 hours (n=2; N=2 per cell line per condition). (**l**) Viability of ERMS cells, evaluated by WST assay, after 48 hours treatment with SAG1.3 (n=12; N=4). **P*<0.05, ***P*<0.01, ****P*<0.001, *****P*<0.0001. Data represent mean ± S.D. PDX – Patient-derived xenografts

**Figure 2.** Single cell cloning and effect of GLI1 over-expression on ERMS cells. (**a**) Representative images of bulk RD cells and three clonal cultures established from single cells immunostained for GLI1 expression. All images were taken at 200x magnification. Scale bar represents 50µm. (**b**) Western Blotting to assess overall GLI1 protein expression level in single cell clones compared to bulk RD cells. (**c**) Sphere forming ability of single cell clones relative to bulk cell line (N=2). The expression of target genes upon stable over-expression of GLI1 (pCMV-GLI1) in RD (**d**; n=10, N=5) and RH36 (**e**; n=12, N=4) compared to control cells (pCMV-Empty; set at baseline). Log_10_ scale. (**f**) Right panel: Colony forming ability of RD cells stably over-expressing GLI1 relative to empty vector transfected cells (N=4). Left panel: Representative images of the colonies stained with Crystal Violet in one well of a standard 6-well plate. (**g**) Western blotting for indicated proteins using lysates of xenografts from stable cell lines. **P*<0.05, ***P*<0.01, ****P*<0.001, *****P*<0.0001. Data represent mean ± S.D. ns - not significant

**Figure 3.** Immunohistochemical analysis of ERMS xenografts. Representative images of immunohistochemistry performed on formalin-fixed paraffin-embedded (FFPE) sections of ERMS xenografts grown in NOD/SCID mice. The images for H&E (Hematoxylin and Eosin), Myogenin and Desmin images were taken at 200x magnification. Scale bar represents 50µm. The images for GLI1 staining were taken at 400x magnification. Scale bar represents 20µm. The presence of a few rhabdomyoblasts was noted within the xenografts. The xenografts had low differentiation with no visible strap cells. GLI1 staining was found to be heterogeneous within xenografts and the over-expression was more readily detected in case of RD. The RH36 pCMV-GLI1 xenograft contained more number of strongly positive GLI1 cells and in general the tumor appeared to be composed of more primitive looking cells.

**Figure 4.** Effect of hedgehog inhibition by synthetic small molecules on ERMS cells. (**a**) Sphere initiation for RH36 cells treated with small-molecule inhibitors GDC-0449 or GANT61 every 48h (3 rounds) during primary sphere formation and further plated for secondary sphere formation in normal media (n=6, N=2). # No spheres formed. **§** No viable cells were recovered for secondary sphere formation. (**b**) GLI1 protein expression is decreased upon siRNA-mediated knockdown in RD adherent cells. (**c**) Left panel: Sphere formation measured following siRNA (10nM) mediated GLI1 knockdown in RH36 adherent cells (N=2). Right panel: GLI1 protein reduction upon siRNA-mediated knockdown in RH36 adherent cells. (**d**) Sphere initiation of RH36 adherent cells pre-treated with hedgehog inhibitors (48 hours) (n=9, N=3 for GDC-0449 and GANT61; n=6, N=2 for LDE-225). Cell cycle profiles generated by flow cytometry-based measurement of Propidium Iodide staining of RD (**e**) and RH36 (**f**) cells treated with hedgehog inhibitors for 48 hours (n=2, N=2 per cell line per condition). Viability evaluated by WST assay, of RD (**g**) and RH36 (**h**) cells after 48 hours treatment with hedgehog inhibitor (n=12, N=4 for GDC-0449 and GANT61; n=6, N=2 for LDE-225). (**i**) Western blotting for GLI1 in RD adherent cells treated with 3µM GANT61 (48h). (**j**) Representative whole mounts of xenograft tumors formed by injecting RD cells either pre-treated with vehicle or GANT61 (3µM) for 48 hours *in vitro*. (**k**) Representative images of immunohistochemistry performed on FFPE sections of xenograft tumors formed by injecting RD cells either pre-treated with vehicle or GANT61 (3µM). The xenografts from GANT61 pre-treatment have fewer GLI1 strongly positive cells and lower desmin positivity. The images for H&E, Myogenin, Desmin and MIB1 were taken at 200x magnification. Scale bar represents 50µm. The images for GLI1 staining were taken at 400x magnification. Scale bar represents 20µm. **P*<0.05, ***P*<0.01, ****P*<0.001. Data represent mean ± S.D.

**Figure 5.** Effect of stable genetic hedgehog inhibition on ERMS cells. mRNA expression of hedgehog target genes in RD (**a**; n=6, N=2) and RH36 (**b**; n=3, N=1) cells over-expressing SUFU (pCMV-SUFU) relative to empty vector control (pCMV-Empty), assessed by qPCR. mRNA expression of hedgehog target genes in RD (**c**; n=9, N=3) and RH36 (**d**; n=9, N=3) cells with stable knockdown of SMO (shSMO) relative to emtpy vector control (pLKO.1), assessed by qPCR. (**e**) Western blotting for indicated proteins in control and stable SMO knockdown ERMS cell lines (**f**) Right panel: Colony forming ability of pCMV-SUFU RD cells relative to pCMV-Empty cells (N=2). Left panel: Representative images of the Crystal Violet stained colonies in one well of a standard 6-well plate. (**g**) Right panel: Colony forming ability of shSMO RD cells relative to pLKO.1 cells (N=2). Left panel: Representative images of the Crystal Violet stained colonies formed in one well of a standard 6-well plate. (**h** and **i**) Cell proliferation measured by BrdU incorporation in RD cells (n=6, N=2). (**j**) Histogram plots of cell cycle profiles generated from Propidium Iodide (PI) staining of RD stable lines by flow cytometry. (**k** and **l**) Quantification of cell cycle stage distributions observed in RD cell lines with different hedgehog pathway status (n=2, N=2 per cell line). (**m** and **n**) Quantification of cell cycle stage distributions observed in RH36 cell lines with different hedgehog pathway status (n=2, N=2 per condition). **P*<0.05, ****P*<0.001. Data represent mean ± S.D. ns – not significant.

**Figure 6.** Cell autonomous ligand-based hedgehog pathway activation in ERMS. Relative mRNA expression level of hedgehog ligands in ERMS sphere cells (**a**: RH36; n=9, N=3 and **b**: RD; n=6, N=2) compared to adherent cells (**a**: RH36; n=9, N=3 and **b**: RD; n=9, N=3). Absolute RNA expression level of hedgehog ligands in ERMS xenografts (**c**: RH36; **d**: RD; n=12, N=4 per cell line) and their respective *in vitro* cultured cells (n=9, N=3 per cell line) normalized to housekeeping gene *HMBS*. (**e** and **f**) Absolute RNA expression level of hedgehog ligands in ERMS PDX samples when grown as tumors (“in vivo”) and when taken into culture as adherent cells (“in vitro”) normalized to HMBS (n=6; N=2 per condition per PDX sample). (**g**, **h** and **i**) QPCR for indicated hedgehog ligands performed on nine ERMS patient tumor samples. Data normalized to geometric mean of *HMBS* and *GAPDH*. (**j**) Scatter plot depicting data from panels **g**, **h** and **i** (excluding patient TB271). Each data point represents a single patient and the line represents median expression level in the cohort. Species-specific ligand RNA expression estimated in ERMS cell line-derived xenografts (**k**: RD; n=12, N=4 per condition and **l**: RH36; n=48, N=16 for human-specific probes; n=30, N=10 for mouse-specific probes). Expression of *Dhh* is higher within ERMS tumors (**m**: RD; n=12, N=4 and **n**: RH36; n=30, N=10) compared to normal murine skeletal muscle (n=6; N=2). Expression levels are normalized to *Gapdh*. **P*<0.05. *P*-value for **k** and **l** from Mann-Whitney test. Data represent mean ± S.D. FeSkM_P - Pool of Fetal Skeletal Muscle RNA (3 donors); AdSkM_P - Pool of Adult Skeletal Muscle RNA (5 donors); h - human; m – mouse; N.E. – Not Expressed, ns – not significant.

**Figure 7.** Effect of hedgehog pathway modulation on ERMS chemoresistance and differentiation. (**a**) Sphere initiation ability of RD cells treated for 48 hours with SN-38 (active metabolite of Irinotecan). Post-treatment equal number of viable cells were counted and plated at clonal density (n=9, N=3). (**b**) Sphere initiation ability of RD cells treated for 48 hours with SN-38 alone or in combination with SMO inhibitor LDE-225 (n=8, N=2). (**c** and **e**) Representative images of RH36 cells stained for PAX7 and MYOGENIN expression. (**d**) Representative images of RD cells stained for PAX7 and MYOGENIN expression. All images were taken at 400x magnification. Scale bar represents 20µm. Intra-cellular flow cytomtery for PAX7 or MYOGENIN positivity performed on RH36 cells treated for 48 hours with indicated concentrations of hedgehog inhibitors GDC-0449 (**f**), GANT61 (**g**) or agonist SAG1.3 (**h**) (n=2, N=2 per condition). (**i**) Percentage of PAX7- or MYOGENIN-positive RD cells treated for 48hrs with 3μM GANT61, normalized to DAPI stained nuclei counted per viewing field using ImageJ and made relative to vehicle treated cells (n=7). (**j**) The mRNA expression of indicated genes in RD cells after 6 days of treatment with GANT61, normalized to HMBS and made relative to expression in vehicle treated cells. The drug was replaced after 72 hours (n=3). **P*<0.05, ***P*<0.01, ****P*<0.001. Data represent mean ± S.D.

**Figure 8.** Effect of hedgehog pathway modulation on ERMS cell motility. Representative images of RD cells stained with Crystal Violet post-invasion (**a**) and post-migration (**b**) in trans-well assay. All images were taken at 200x magnification. Scale bar represent 50µm.

**Figure 9.** Confirmation of qPCR screen hit. NANOG RNA expression analysis using Taqman-based qPCR in pCMV-GLI1 (**a**: n=12, N=4), shSMO (**b**: n=9, N=3) and pCMV-SUFU (**c**: n=6, N=2) ERMS cells relative to their respective control cell lines. (**d**) Western blotting for NANOG protein expression (arrow) in control and SUFU-overexpressing ERMS cells. (**e**) Representative images of RH36 stable cells co-immunostained for GLI1 and NANOG expression. (**f**) Representative images of primary cells from ERMS PDX samples co-immunostained for GLI1 and NANOG expression. All images (**e** and **f**) were taken at 400x magnification. Scale bar represents 20µm. **P*<0.05. Data represent mean ± S.D.

**Figure 10.** Modulation of NANOG expression by hedgehog pathway activation. (**a**) NANOG expressing RH36 cells counted after 48hrs treatment with SAG1.3 (n=3). Representative images for RD (**b**) and RH36 (**c**) cells treated with SAG1.3 and stained for NANOG. All images were taken at 400x magnification. Scale bar represents 20µm. (**d**) NANOG RNA expression, normalized to HMBS, in RD xenografts (n=6, N=2) and adherent cultures (n=9, N=3). (**e**) NANOG expression in RH36 xenografts and adherent cells. Left panel: NANOG RNA expression normalized to HMBS in xenografts (n=6, N=2) and adherent cells (n=9, N=3). Right panel: Western blot showing expression of indicated proteins. (**f**) NANOG RNA expression in ERMS sphere cells relative to adherent cultures (n=6, N=2). (**g**) NANOG protein expression level (arrow) in ERMS adherent and sphere cells. **P*<0.05, ***P*<0.01. Data represent mean ± S.D.

**Figure 11.** Characterization of phenotype rescue cell lines and additional clinical data analysis. (**a**) Left panel: RNA expression analysis of indicated genes in RD cells with stable NANOG knockdown (shNANOG) relative to control (pLKO.1) (n=6, N=2). Right panel: Western Blot analysis of indicated proteins. (**b**) Left panel: RNA analysis of indicated genes upon siRNA-mediated NANOG knockdown (10nM; 48 hours) in RD adherent cells (n=6; N=2). Right panel: Western Blot analysis of indicated proteins upon siRNA-mediated NANOG knockdown in RD adherent cells. ***P*<0.01, ****P*<0.001 from Student’s t-test. (**c**) Western Blot analysis of RD cells for indicated proteins upon transient overexpression of NANOG. Expression analysis to verify the stable cell lines generated from RD (**d**) and RH36 (**e**) cells with knockdown of NANOG in the presence of GLI1 over-expression (n=6; N=2). Data represent mean ± S.D. (**f**) Representative images of immunohistochemistry performed on FFPE sections of RH36 stable line xenografts grown in NOD/SCID mice. The images were taken at 200x magnification. Scale bar represents 50µm. (**g**) Western blot analysis of indicated proteins in RH36 xenografts. (**h**) Representative images of immunohistochemistry staining for GLI1 and NANOG within an ERMS patient tumor core. All images were taken at 400x magnification. Scale bar represents 20µm. Kaplan-Meier curve for event-free survival of 91 ERMS patients determined to be either negative (black line) or positive (grey line) for expression of GLI1 (**i**) or NANOG (**j**) alone. The *P*-values were generated using log-rank test.
