# Supplementary material for: Targeting hedgehog signaling reduces self-renewal in embryonal rhabdomyosarcoma
Source: Oncogene. 2015 Jul 20;35(16):2020–30. doi: 10.1038/onc.2015.267 (PMC5399168; doi:10.1038/onc.2015.267)
Supplement: Supplementary Table 1 [file onc2015267x2.doc]

**Supplementary Table 1.** List of TaqMan®-probe based gene expression assays used in the study.

| **Gene** | **Species** | **TaqMan® Gene Expression Assay ID** |
| --- | --- | --- |
| HMBS | Human | Hs00609297_m1 |
| GAPDH | Human | Hs02758991_g1 |
| GLI1 | Human | Hs01110766_m1 |
| GLI2 | Human | Hs01119974_m1 |
| GLI3 | Human | Hs00609233_m1 |
| PTCH1 | Human | Hs00181117_m1 |
| HHIP | Human | Hs01011008_m1 |
| SMO | Human | Hs01090242_m1 |
| SUFU | Human | Hs00171981_m1 |
| SHH | Human | Hs00179843_m1 |
| IHH | Human | Hs00745531_s1 |
| DHH | Human | Hs00368306_m1 |
| NANOG | Human | Hs02387400_g1 |
| PDGFRA | Human | Hs00998018_m1 |
| CKM | Human | Hs00176490_m1 |
| MYL1 | Human | Hs00984899_m1 |
| Shh | Mouse | Mm00436528_m1 |
| Dhh | Mouse | Mm01310203_m1 |
| Ihh | Mouse | Mm00439613_m1 |
| Gapdh | Mouse | Mm99999915_g1 |
