# Supplementary material for: Targeting hedgehog signaling reduces self-renewal in embryonal rhabdomyosarcoma
Source: Oncogene. 2015 Jul 20;35(16):2020–30. doi: 10.1038/onc.2015.267 (PMC5399168; doi:10.1038/onc.2015.267)
Supplement: Supplementary Table 2 [file onc2015267x3.doc]

| **Primary antibodies (Anti-human)^1^** | | | | | | |
| --- | --- | --- | --- | --- | --- | --- |
| **Detected protien (clone)** | **Catalogue #** | **Company** | **Species** | **Clonality** | **Application** | **Dilution** |
| GLI1 (V812) | 2534 | Cell Signaling Technology | Rabbit | poly | WB | 1/1000 |
| HHIP (R-20) | sc-9408 | Santa Cruz | Goat | poly | WB | 1/100 |
| SUFU  (C81H7) | 2522 | Cell Signaling Technology | Rabbit | mono | WB | 1/1000 |
| SMO (N-19) | sc-6366 | Santa Cruz | Goat | poly | WB | 1/500 |
| PTCH1  (H-267) | sc-9016 | Santa Cruz | Rabbit | poly | WB | 1/500 |
| NANOG | ab21624 | Abcam | Rabbit | poly | WB | 1/500 |
| NANOG (D73G4) | 4903 | Cell Signaling Technology | Rabbit | mono | WB | 1/1000 |
| GAPDH (D16H11) | 5174 | Cell Signaling Technology | Rabbit | mono | WB | 1/10000 |
| α-TUBULIN (DM1A) | T9026 | Sigma-Aldrich | Mouse | mono | WB | 1/40000 |
| PAX7 | PAX7 | Developemtal Studies Hybridoma Bank | Mouse | mono | IF and IC-FC | 1/50 |
| MYOGENIN (M-225) | sc-576 | Santa Cruz | Rabbit | poly | IF and IC-FC | 1/1000 |
| GLI1 (H-300) | sc-20687 | Santa Cruz | Rabbit | poly | IF | 1/100 |
| NANOG (hNanog.2) | 14-5768-82 | eBioscience | Mouse | mono | IF | 1/50 |
| MYOGENIN (L026) | PA0226 | Novocastra, Leica | Mouse | mono | IHC | 1/20 |
| DESMIN (D33) | M076029 | Dako | Mouse | mono | IHC | 1/20 |
| MIB-1 (30-9) | - | Ventana, Roche | Rabbit | mono | IHC | Prediluted (Ventana, Roche) |
| GLI1 (H-300) | sc-20687 | Santa Cruz | Rabbit | poly | IHC | 1/75 |
| NANOG  (NNG-811) | ab62734 | Abcam | Mouse | mono | IHC | 1/2000 |
| **Secondary Antibodies^2^** | | | | | | |
| **Detected species** | **Catalogue no.** | **Company** | **Species** | **Conjugation** | **Application** | **Dilution** |
| Anti-Mouse IgG | 7076 | Cell Signaling Technology | Horse | HRP | WB | 1/2000 |
| Anti-Rabbit IgG | 7074 | Cell Signaling Technology | Goat | HRP | WB | 1/2000 |
| Anti-Goat IgG | A50-201P | Bethyl | Donkey | HRP | WB | 1/2000 |
| Anti-Mouse IgG | A21202 | Life technologies | Donkey | Alexa Fluor-488 | IF and IC-FC | 1/500 |
| Anti-Rabbit IgG | A21206 | Life technologies | Donkey | Alexa Fluor-488 | IF and IC-FC | 1/500 |
| Anti-Mouse IgG | A21203 | Life technologies | Donkey | Alexa Fluor-594 | IF | 1/500 |
| Anti-Rabbit IgG | A21207 | Life technologies | Donkey | Alexa Fluor-594 | IF | 1/500 |

**Supplementary Table 2.** Details of antibodies used in the study

^1^ Abbreviations: WB – Western Blotting, IF – Immunofluorescence, IC-FC – Intra-cellular Flow Cytometry, IHC – Immunohistochemistry, HRP – Horseradish peroxidise

^2^ HRP-conjugated secondary antibodies for IHC were purchased pre-diluted in the reagents associated with the automated systems used for processing (see text).
