# Supplementary figures and images for: Targeting hedgehog signaling reduces self-renewal in embryonal rhabdomyosarcoma
Source: Oncogene. 2015 Jul 20;35(16):2020–30. doi: 10.1038/onc.2015.267 (PMC5399168; doi:10.1038/onc.2015.267)

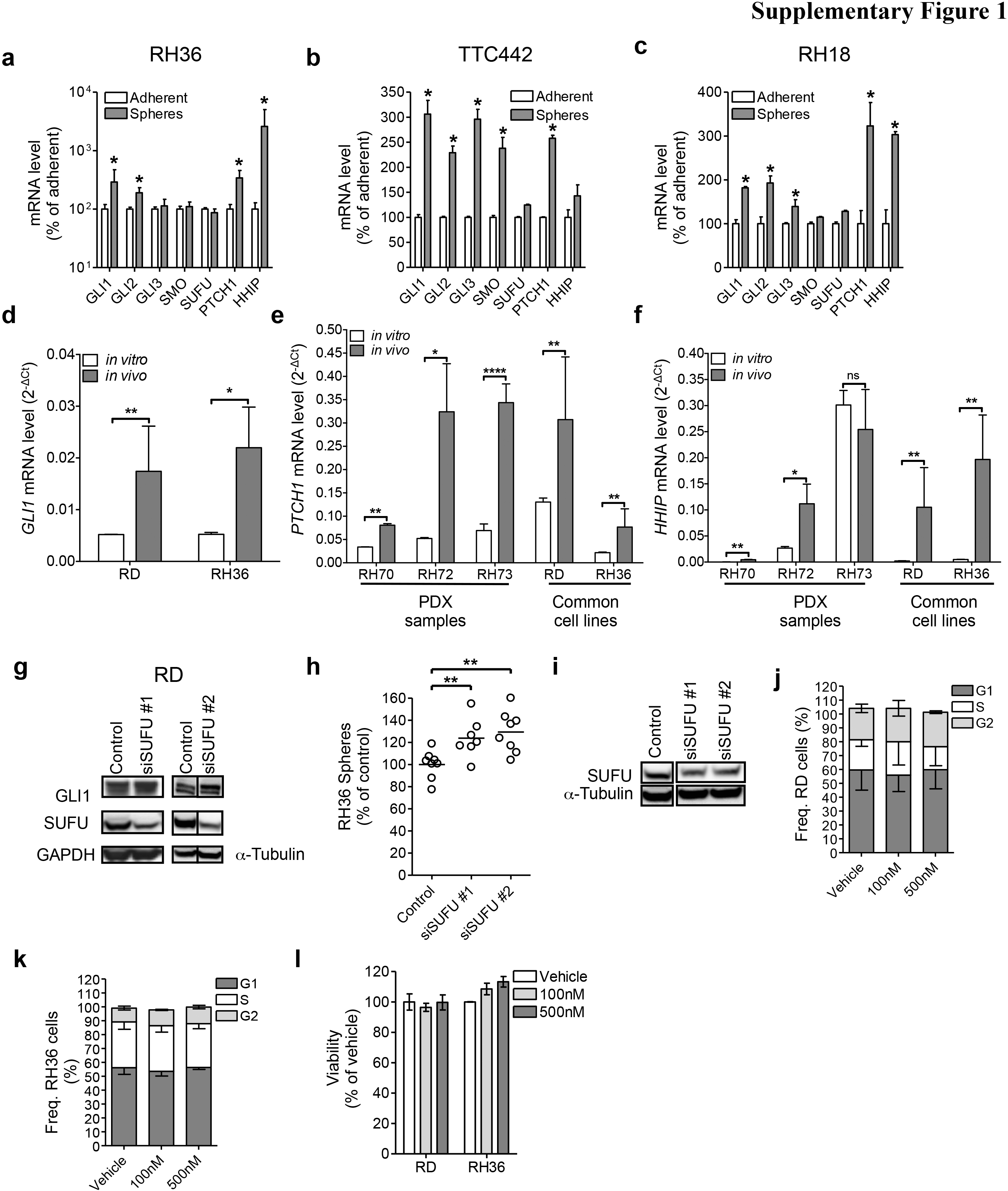

Supplement: Supplementary Figure 1 [file onc2015267x4.tif]

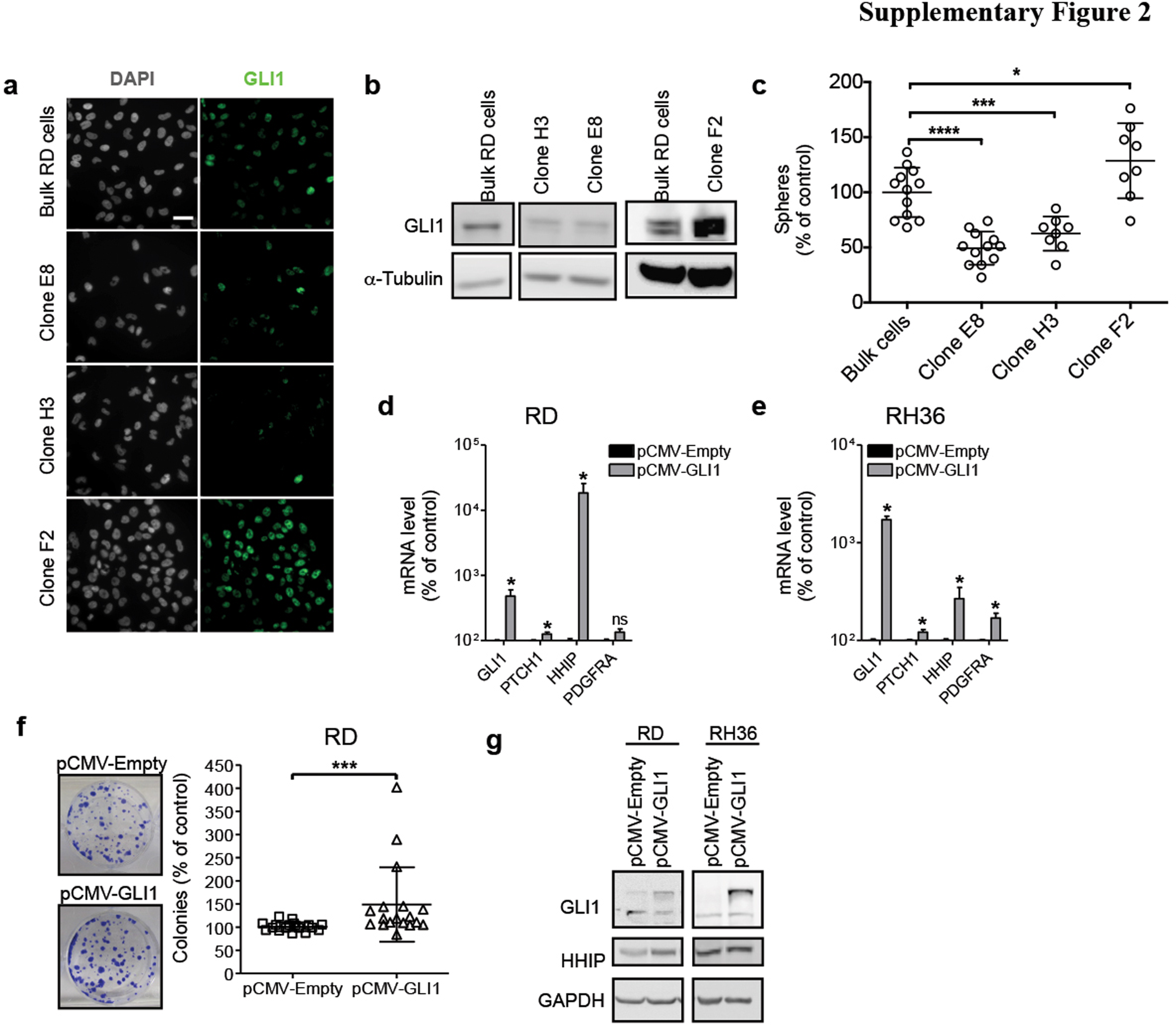

Supplement: Supplementary Figure 2 [file onc2015267x5.tif]

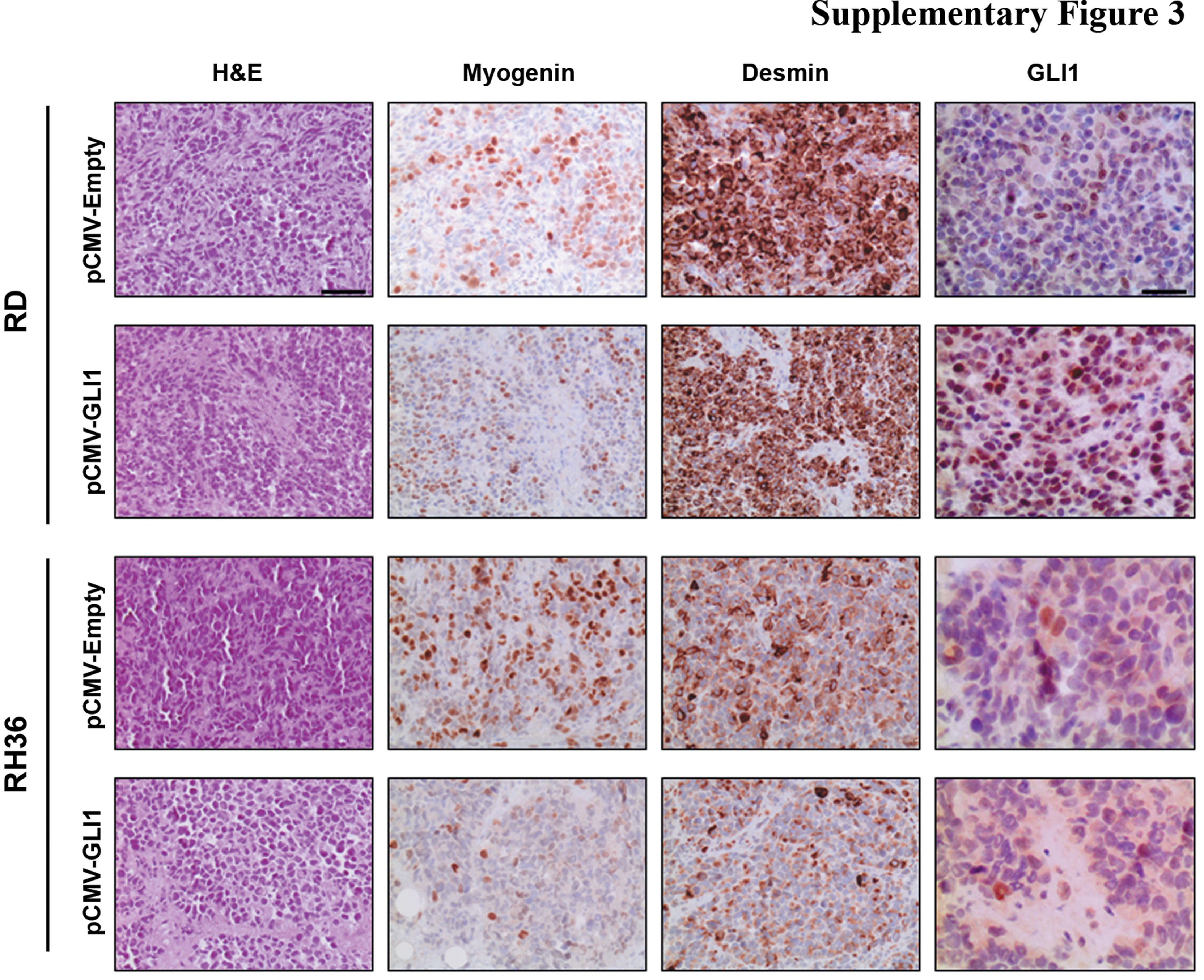

Supplement: Supplementary Figure 3 [file onc2015267x6.tif]

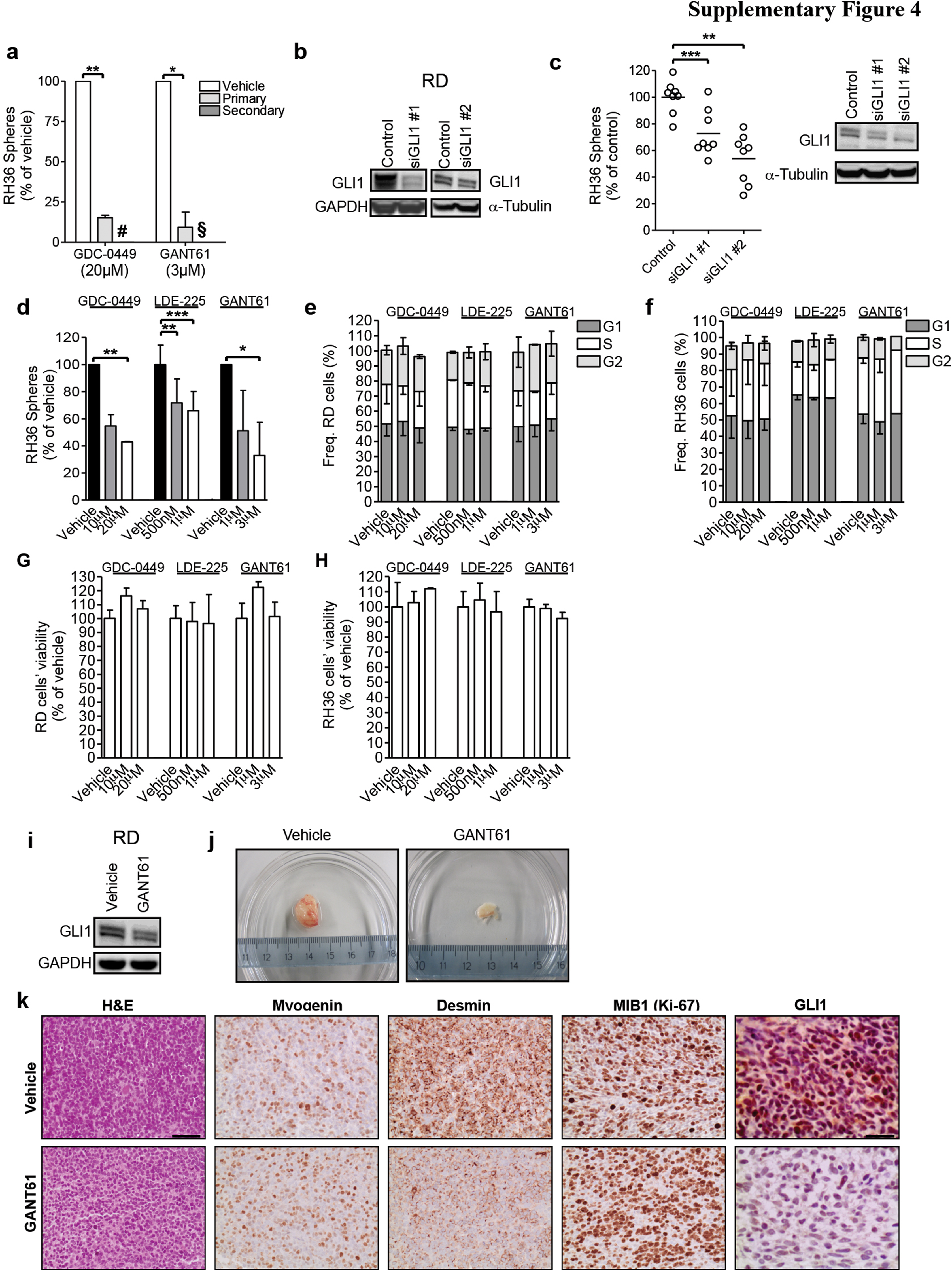

Supplement: Supplementary Figure 4 [file onc2015267x7.tif]

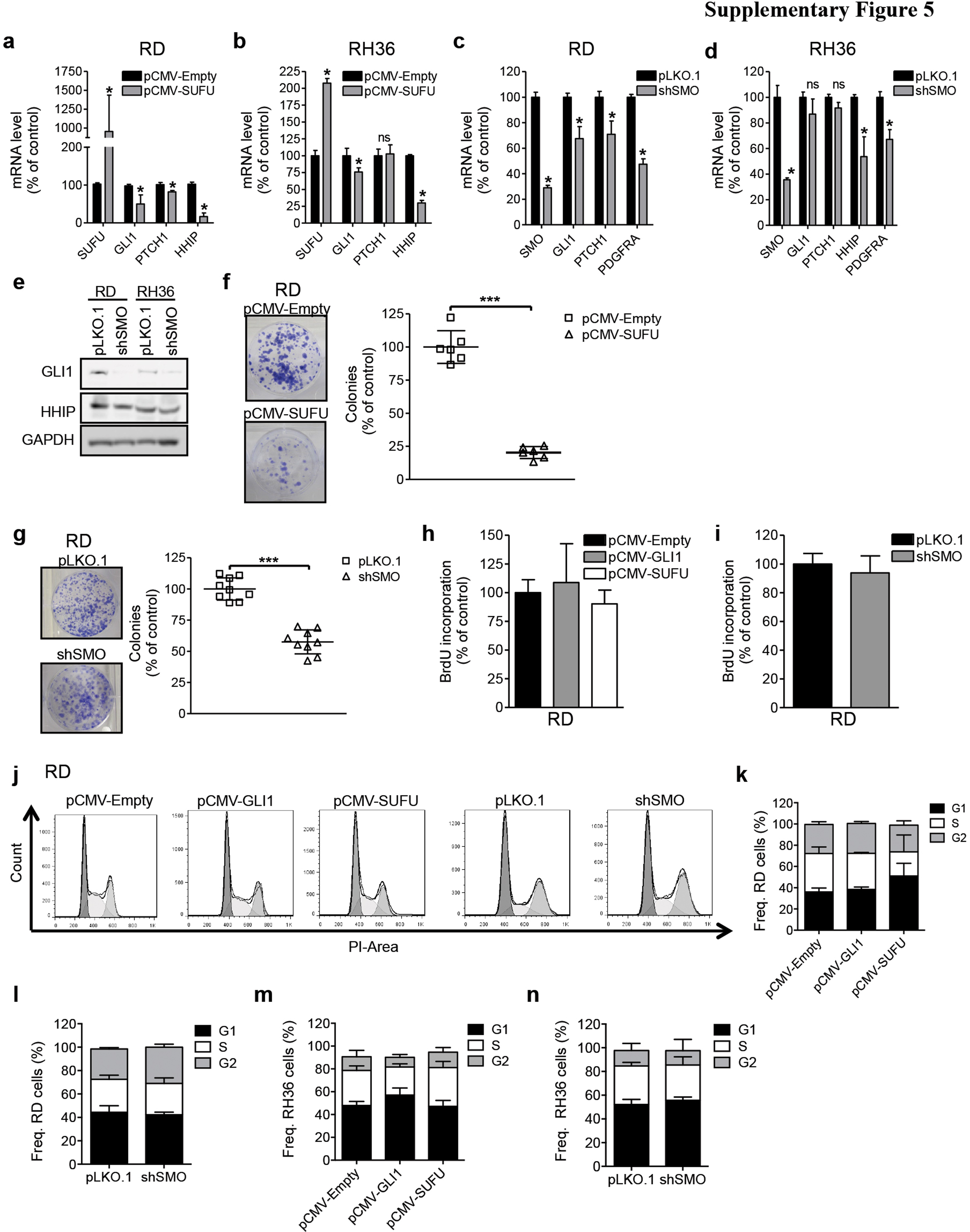

Supplement: Supplementary Figure 5 [file onc2015267x8.tif]

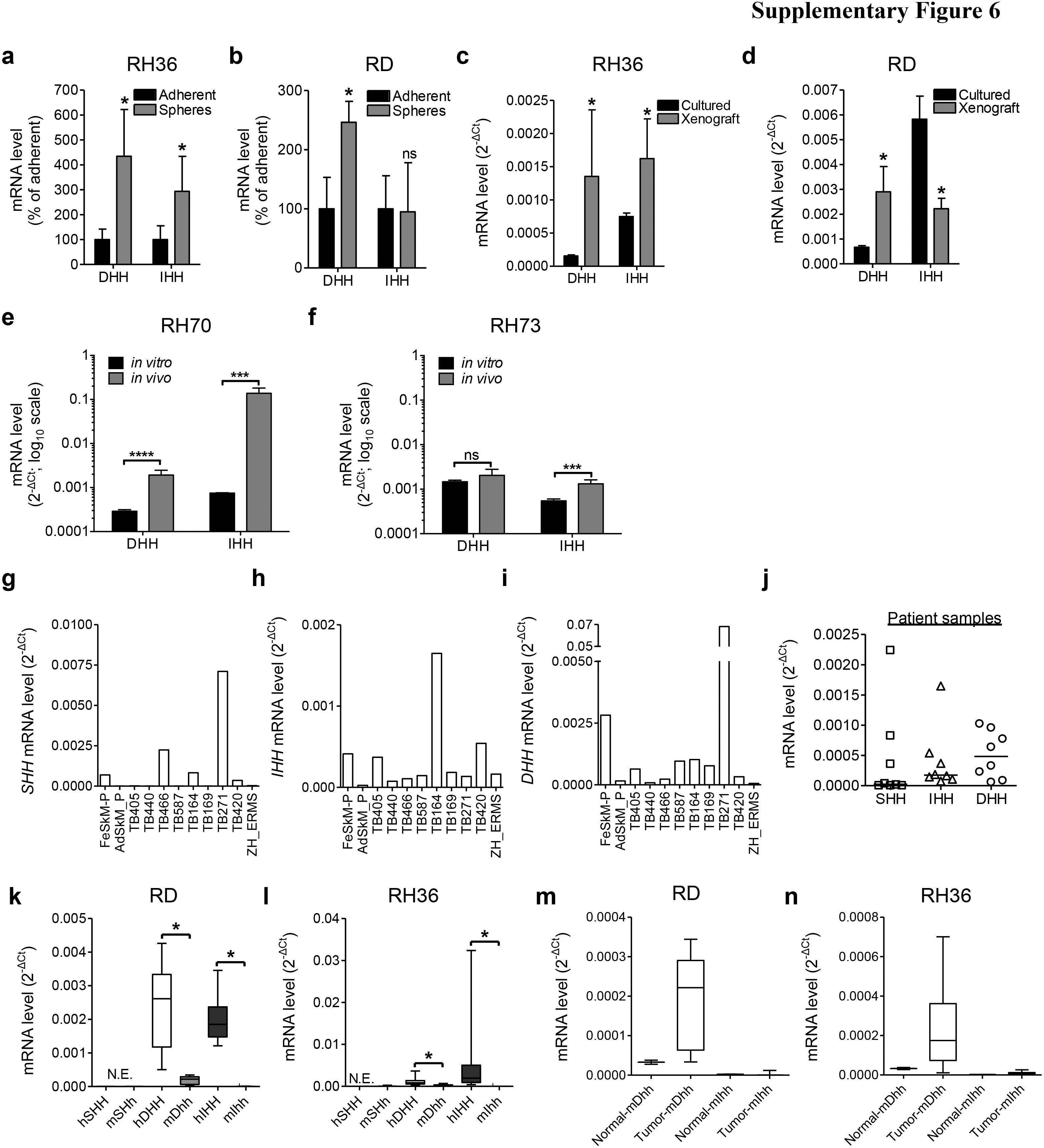

Supplement: Supplementary Figure 6 [file onc2015267x9.tif]

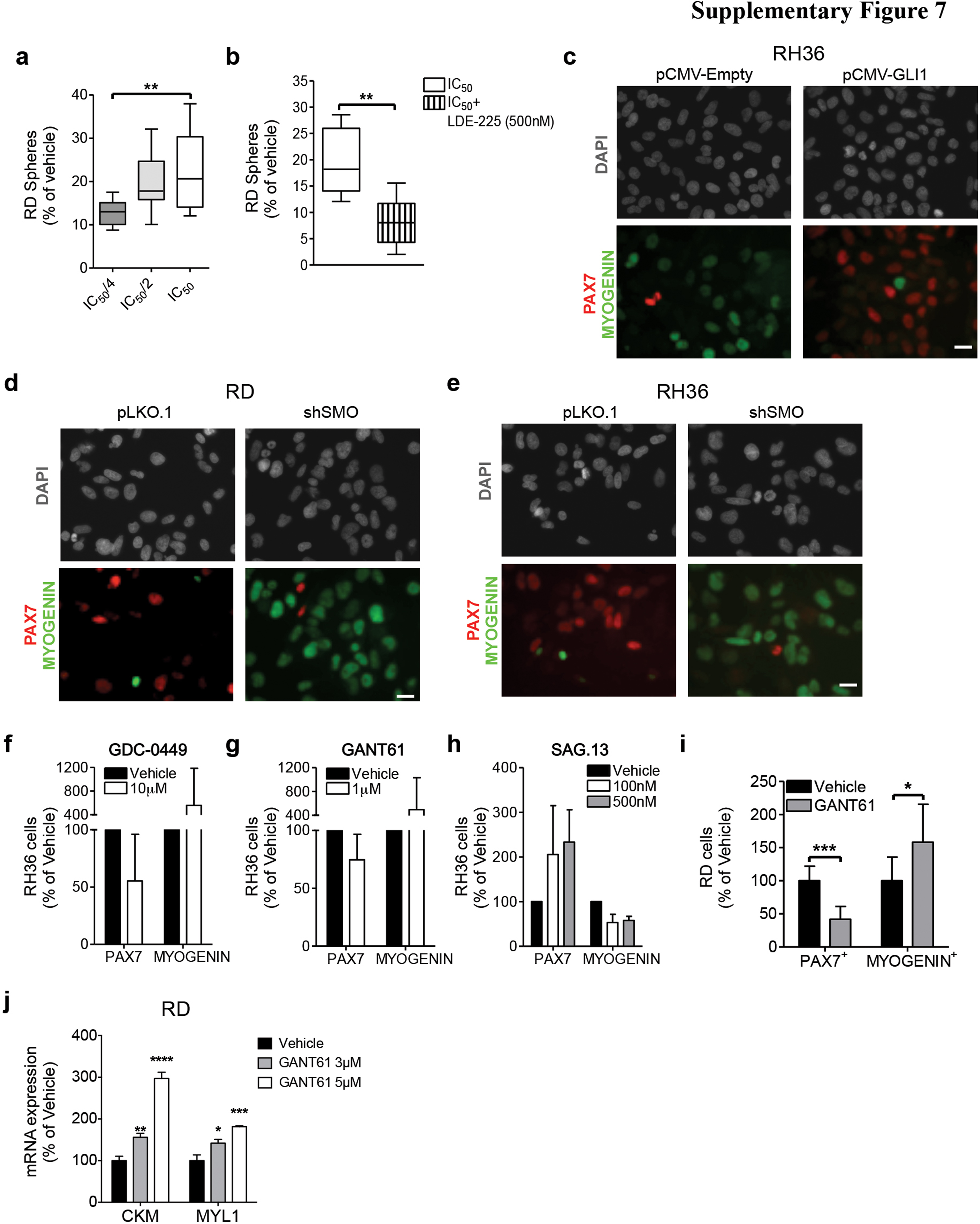

Supplement: Supplementary Figure 7 [file onc2015267x10.tif]

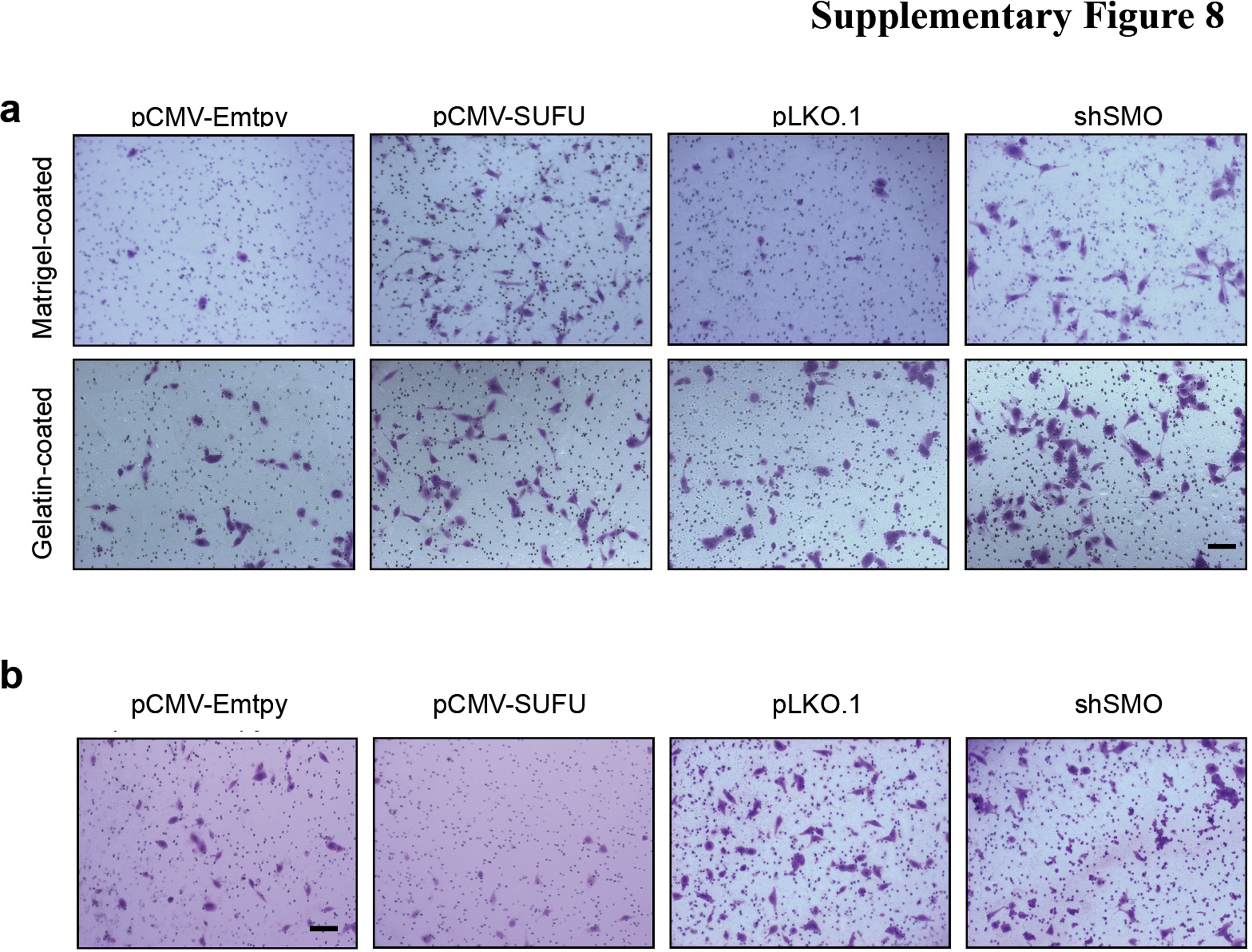

Supplement: Supplementary Figure 8 [file onc2015267x11.tif]

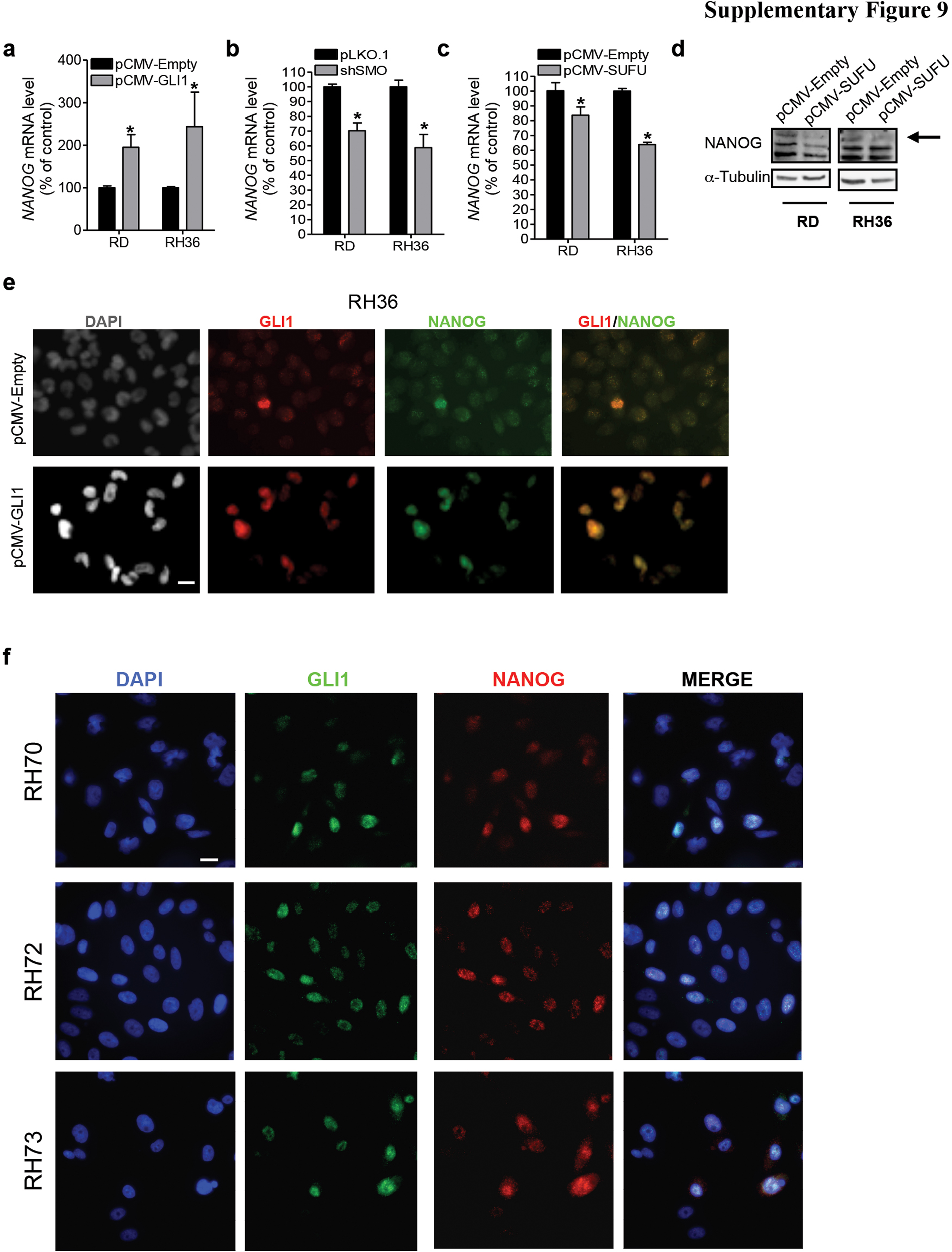

Supplement: Supplementary Figure 9 [file onc2015267x12.tif]

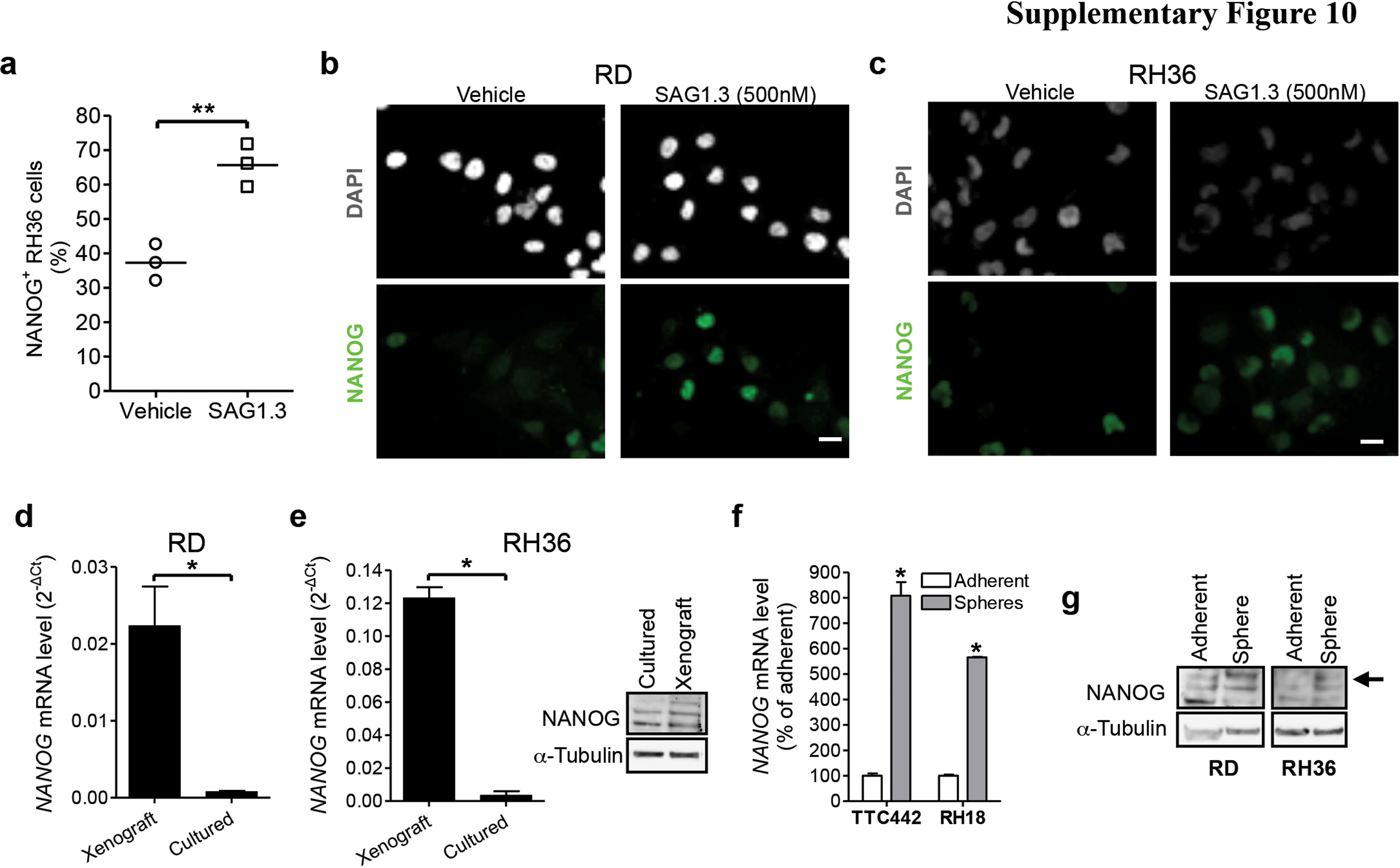

Supplement: Supplementary Figure 10 [file onc2015267x13.tif]

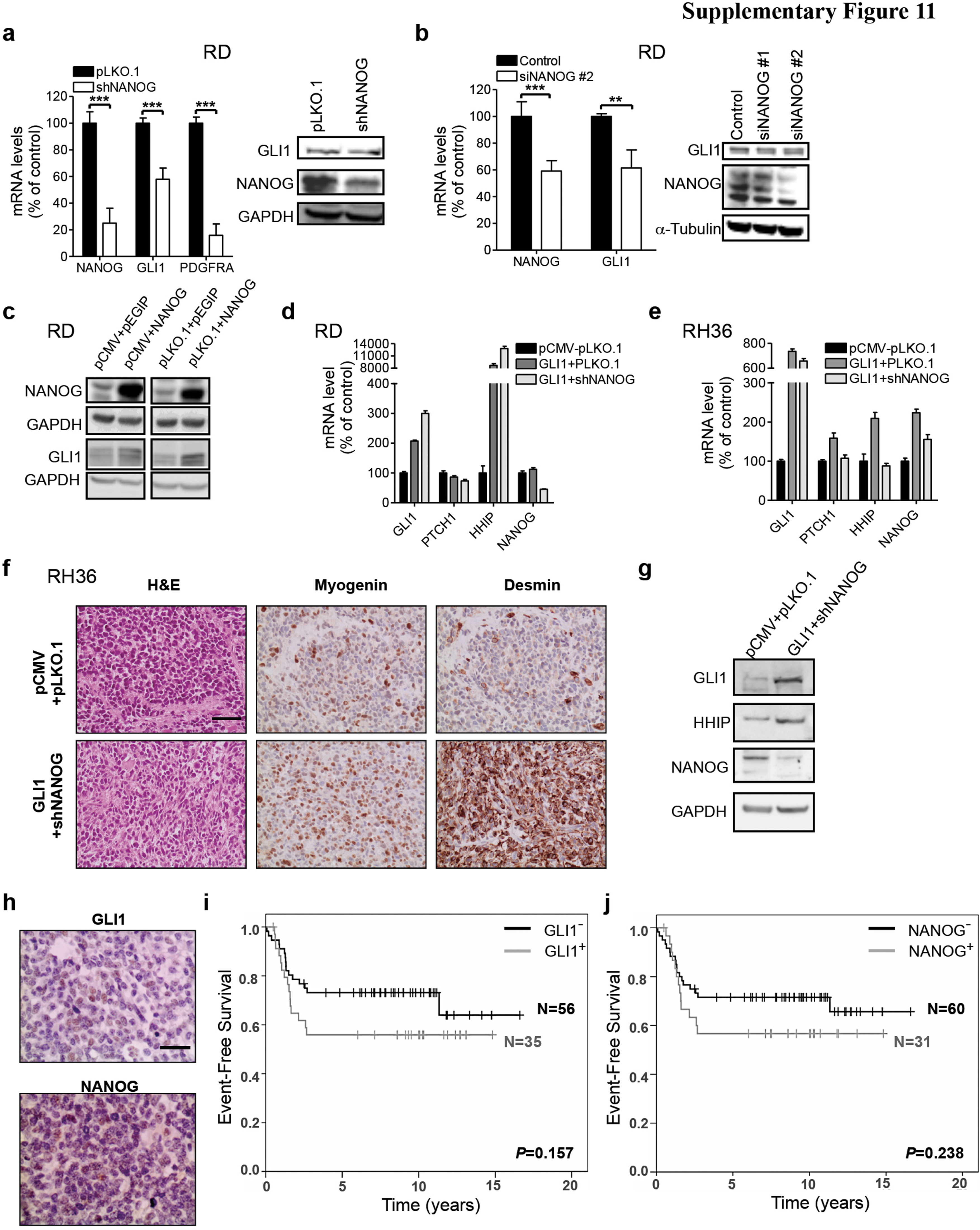

Supplement: Supplementary Figure 11 [file onc2015267x14.tif]
